# Structural Insight into Substrate Specificity of 3-Hydroxypropionyl-Coenzyme A Dehydratase from *Metallosphaera sedula*

**DOI:** 10.1038/s41598-018-29070-w

**Published:** 2018-07-16

**Authors:** Donghoon Lee, Kyung-Jin Kim

**Affiliations:** 1 KNU Creative BioResearch Group, School of Life Sciences and Biotechnology, Kyungpook National University, Daegu, 41566 Republic of Korea; 2 KNU Institute for Microorganisms, Kyungpook National University, Daegu, 41566 Republic of Korea

## Abstract

*Metallosphaera sedula* is a thermoacidophilic autotrophic archaeon known to utilize the 3-hydroxypropionate/4-hydroxybutyrate cycle (3-HP/4-HB cycle) as carbon fixation pathway. 3-Hydroxypropionyl-CoA dehydratase (3HPCD) is an enzyme involved in the 3-HP/4-HB cycle by converting 3-hydroxypropionyl-CoA to acryloyl-CoA. To elucidate the molecular mechanism of 3HPCD from *M. sedula* (*Ms*3HPCD), we determined its crystal structure in complex with Coenzyme A (CoA). *Ms*3HPCD showed an overall structure and the CoA-binding mode similar to other enoyl-CoA hydratase (ECH) family enzymes. However, compared with the other ECHs, *Ms*3HPCD has a tightly formed α3 helix near the active site, and bulky aromatic residues are located at the enoyl-group binding site, resulting in the enzyme having an optimal substrate binding site for accepting short-chain 3-hydroxyacyl-CoA as a substrate. Moreover, based on the phylogenetic tree analysis, we propose that the 3HPCD homologues from the phylum *Crenarchaeota* have an enoyl-group binding pocket similar to that of bacterial short-chain ECHs.

## Introduction

Due to the increase in carbon dioxide (CO_2_) emissions, environmental problems such as climate change have attracted much attention around the world, and many countries are making efforts to reduce CO_2_ emissions with climate change measures^1–3^. As a result, there is a growing interest in microbial strains capable of carbon fixation. In prokaryotes, six distinct autotrophic CO_2_ fixation pathways have been identified so far: the reductive pentose phosphate cycle (Calvin-Benson-Bassham cycle)^4^, the reductive citric acid cycle (Arnon-Buchanan cycle)^5,6^, the reductive acetyl-CoA pathway (Wood-Ljungdahl pathway)^7^, the 3-hydroxypropionate/malyl-CoA cycle^8^, the dicarboxylate/4-hydroxybutyrate cycle^9^, and the 3-hydroxypropionate/4-hydroxybutyrate (3-HP/4-HB) cycle^10^.

Among the autotrophic CO_2_ fixation pathways, the 3-HP/4-HB cycle is one of the most recently discovered. The thermoacidophilic autotrophic *Crenarchaeota* is known to have the 3-HP/4-HB cycle^11,12^. The archaeal phylum *Crenarchaeota* comprises four orders of hyperthermophilic organisms, namely *Sulfolobales*, *Thermoproteales*, *Desulfurococcales*, and *Caldisphaerales*^13,14^. Based on the sequence information, *Metallosphaera sedula* is one of the S*ulfolobales*, which includes S*ulfolobus* and *Acidianus*^15,16^. *M. sedula* is the most extensively studied thermoacidophilic archaea due to its carbon fixation capability using the 3-HP/4-HB cycle^11,17^. *M. sedula* was originally isolated from Pisciarelli Solfatara in Naples, Italy, which is a volcanic area with very low pH, high temperature, and high metal ion concentrations, and the optimal growth conditions for *M. sedula* is 75 °C and pH 2.0^17,18^.

**Figure 1:**
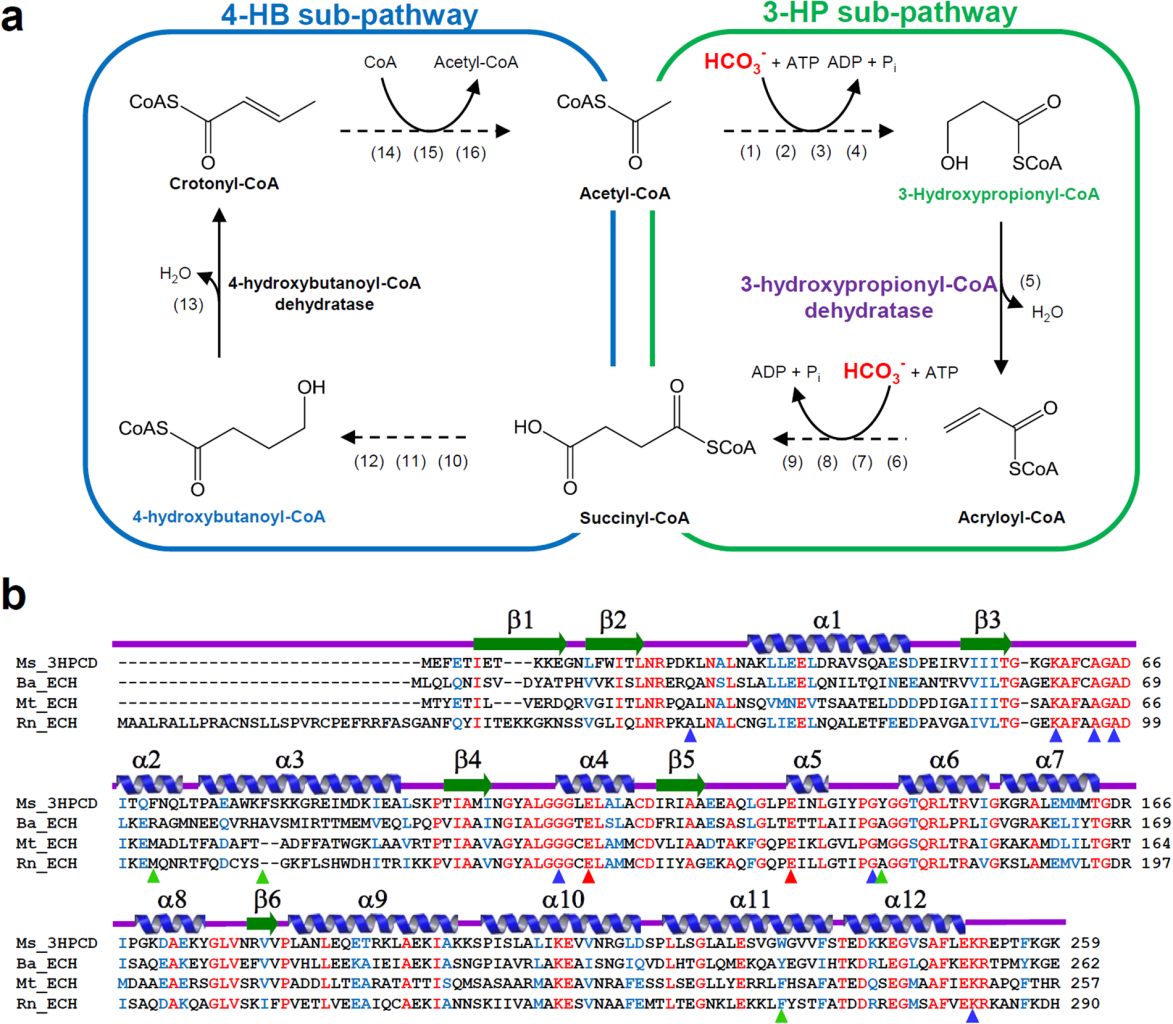
Schematic diagram of the 3-HP/4-HB cycle and amino acid sequence alignment of ECHs. (**a**) Schematic diagram of the 3-HP/4-HB cycle. Enzymes are the following: (1) acetyl-CoA carboxylase, (2) malonyl-CoA reductase (NADPH), (3) malonate semialdehyde reductase (NADPH), (4) 3-hydroxypropionyl-CoA synthetase (AMP forming), (5) 3-hydroxypropionyl-CoA dehydratase, (6) acryloyl-CoA reductase (NADPH), (7) propionyl-CoA carboxylase, (8) methylmalonyl-CoA epimerase, (9) methylmalonyl-CoA mutase, (10) succinyl-CoA reductase (NADPH), (11) succinate semialdehyde reductase (NADPH), (12) 4-hydroxybutyryl-CoA synthetase (AMP forming), (13) 4-hydroxybutyryl-CoA dehydratase, (14) crotonyl-CoA hydratase, (15) (S)-3-hydroxybutyryl-CoA dehydrogenase (NAD^+^), (16) acetoacetyl-CoA β-ketothiolase. (**b**) Amino acid sequence alignment of ECH enzymes. The secondary structure elements are drawn based on the structure of *Ms*3HPCD. Identical and highly conserved residues are presented in red and blue colored characters, respectively. The residues involved in enzyme catalysis, binding of CoA, and formation of the 3-HP binding pocket in *Ms*3HPCD are indicated by red, blue, and green colored triangles, respectively. Ms, Ba, Mt and Rn represent *Metallosphaera sedula*, *Bacillus anthracis*, *Mycobacterium tuberculosis*, and *Rattus norvegicus*, respectively.

The 3-HP/4-HB cycle is largely divided into two sub-pathways, the 3-HP sub-pathway and the 4-HB sub-pathway (Fig. 1a). In the 3-HP sub-pathway, acetyl-CoA is converted to succinyl-CoA by the addition of two molecules of bicarbonate (Fig. 1a). This sub-pathway consists of nine enzymes, including 3-hydroxypropionyl-CoA dehydratase (3-HPCD)^10,12,19–21^. The 4-HB sub-pathway regenerates acetyl-CoA, the first CO_2_ acceptor molecule. In the 4-HB sub-pathway, succinyl-CoA is converted into two molecules of acetyl-CoA through seven enzymatic reactions (Fig. 1a)^10,12,19–21^. One of the two acetyl-CoA is used as a starting material for the 3-HP sub-pathway and the other is used for other pathways such as gluconeogenesis. 3-HPCD catalyzes the dehydration reaction of 3-hydroxypropionyl-CoA (3-HP-CoA) to produce acryloyl-CoA (Fig. 1a)^10,21^. The enzyme belongs to the enoyl-CoA hydratase (ECH) family^21^. Although 3-HPCD has been biochemically studied and characterized^21^, there have been no structural studies reported so far. In this study, we report the first crystal structure of 3-HPCD from *M. sedula* (*Ms*3HPCD) in complex with CoA, and reveal how the enzyme accommodates 3-HP-CoA as a substrate. Through structural comparisons with other ECH enzymes, we also provide insights into how the protein accommodates only short chain hydroxyacyl-CoA as a substrate.

## Results and Discussion

### Overall structure of *Ms*3HPCD

**Figure 2:**
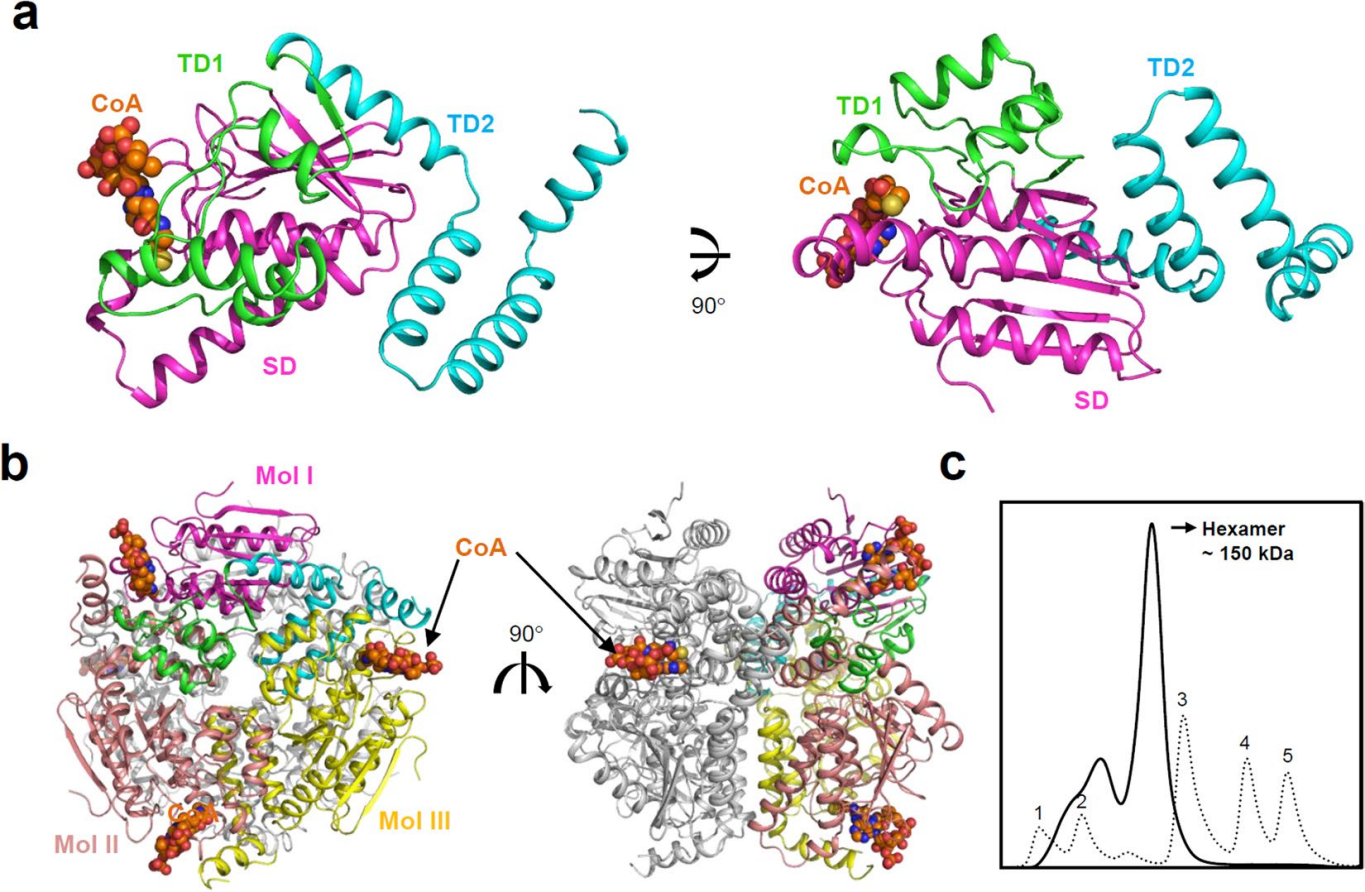
Overall structure of *Ms*3HPCD. (**a**) The monomeric structure of *Ms*3HPCD. The *Ms*3HPCD structure is shown as a cartoon diagram. SD, TD1, and TD2 are distinguished by different colors of magenta, green, and cyan, respectively, and labeled appropriately. The bound CoA molecule is shown as an orange colored sphere. The right figure is the left figure rotated horizontally by 90°. (**b**) Hexameric structure of *Ms*3HPCD. The hexameric structure of *Ms*3HPCD is presented as a cartoon diagram. Mol I is presented with a color scheme same as (**a**). Mol II and III are presented with colors of salmon and yellow, respectively. The other trimer is shown with a grey color. The bound CoA molecule is shown as sphere models with an orange color. The right figure is rotated by 90° vertically from the left figure. (**c**) Size-exclusion chromatography of *Ms*3HPCD. The *Ms*3HPCD and standard samples are distinguished with solid and dotted lines, respectively. 1, 2, 3, 4, and 5 indicate void and standard samples of Ferritin (440 kDa), Conalbumin (75 kDa), Carbonic anhydrase (29 kDa), Ribonuclease A (13.7 kDa), respectively.

To elucidate the molecular mechanism of *Ms*3HPCD, we purified, crystallized, and determined its crystal structure at a 1.8 Å resolution (Table 1). The atomic structure was in good agreement with the X-ray crystallographic statistics for bond angles, bond lengths, and other geometric parameters. The overall structure of *Ms*3HPCD was similar to that of other enoyl-CoA hydratases from *Bacillus anthracis* (*Ba*ECH, PDB code 3KQF), *Mycobacterium tuberculosis* (*Mt*ECH, PDB code 3Q0J), and *Rattus norvegicus* (*Rn*ECH, PDB code 1MJ3, 1DUB, 2DUB) (Fig. 1b)^22^. The monomeric structure of *Ms*3HPCD consists of three distinct domains: One spiral domain and two trimerization domains (Fig. 2a). The spiral domain (SD; Met1-Ala124) comprises five β-sheets and four α-helices, and the five centered β-sheets are surrounded by four α-helices (Fig. 2a). Trimerization domain 1 (TD1; Glu125-Leu184) comprises four α-helices and one β-strand and trimerization domain 2 (TD2; Ala185-Arg252) consists of only four α-helices (Fig. 2a). The active site is formed between two monomers and all three domains are involved in the formation of the active site (Fig. 2b).

**Table 1:** Data collection and structural refinement statistics.

	*Ms*3HPCD_CoA
**Data collection**	
Space group	P21
Cell dimensions	
*a*, *b*, *c* (Å)	84.6, 130.1, 84.7
α, β, γ (°)	90.00, 119.57, 90.00
Resolution (Å)	50.00–1.80 (1.83–1.80)
*R*_sym_ or *R*_merge_	8.2 (35.9)
*I*/σ (*I*)	30.4 (3.05)
Completeness (%)	98.4 (97.1)
Redundancy	5.4 (3.6)
**Refinement**	
Resolution (Å)	50.00–1.80
No. reflections	138405
*R*_work_/*R*_free_	18.3 (22.9)
No. atoms	13087
Protein	11941
Ligand/ion	296
Water	850
*B*-factors	26.948
Protein	26.834
Ligand/ion	54.628
Water	33.414
R.m.s. deviations	
Bond lengths (Å)	0.011
Bond angles (°)	1.499

^a^The numbers in parentheses are statistics from the highest resolution shell.

^b^*R*sym = Σ |Iobs−Iavg|/ Iobs, where Iobs is the observed intensity of individual reflection and *I*avg is average over symmetry equivalents.

^c^*R*work = Σ ||Fo|−|Fc||/Σ |Fo|, where |Fo| and |Fc| are the observed and calculated structure factor amplitudes, respectively. *R*free was calculated with 5% of the data.

The asymmetric unit of the crystal contained six *Ms*3HPCD monomers, corresponding to a hexameric assembly (Fig. 2b). Size-exclusion chromatography confirmed that the enzymes also exists as a hexamer in solution (Fig. 2c). In general, ECH family enzymes function as hexamer^23,24^. The hexamer is formed by dimerization of two trimers. The trimer of *Ms*3HPCD are mainly formed by interactions between TD1 and TD2 (Fig. 2b). TD1 of one monomer (Mol I) interacts with TD1 and TD2 of neighboring monomer (Mol II), and TD2 of one monomer (Mol I) interacts with SD and TD1 of neighboring monomer (Mol III) (Fig. 2b). Each trimer is stabilized by 32 hydrogen bonds and six salt bridges between each monomer. The hexameric structure of *Ms*3HPCD is formed by contact between two trimers, and two α-helices (α10 and α11) of TD2 from each monomer are main contributors of hexamerization (Fig. 2b). PISA software calculated that a total of 36,290 Å^2^ of solvent-accessible interface is buried upon hexamer formation. For dimerization of two trimers, a total of 15,050 and 14,380 Å^2^ of solvent-accessible surface areas per each trimer are buried.

### Active site of *Ms*3HPCD

**Figure 3:**
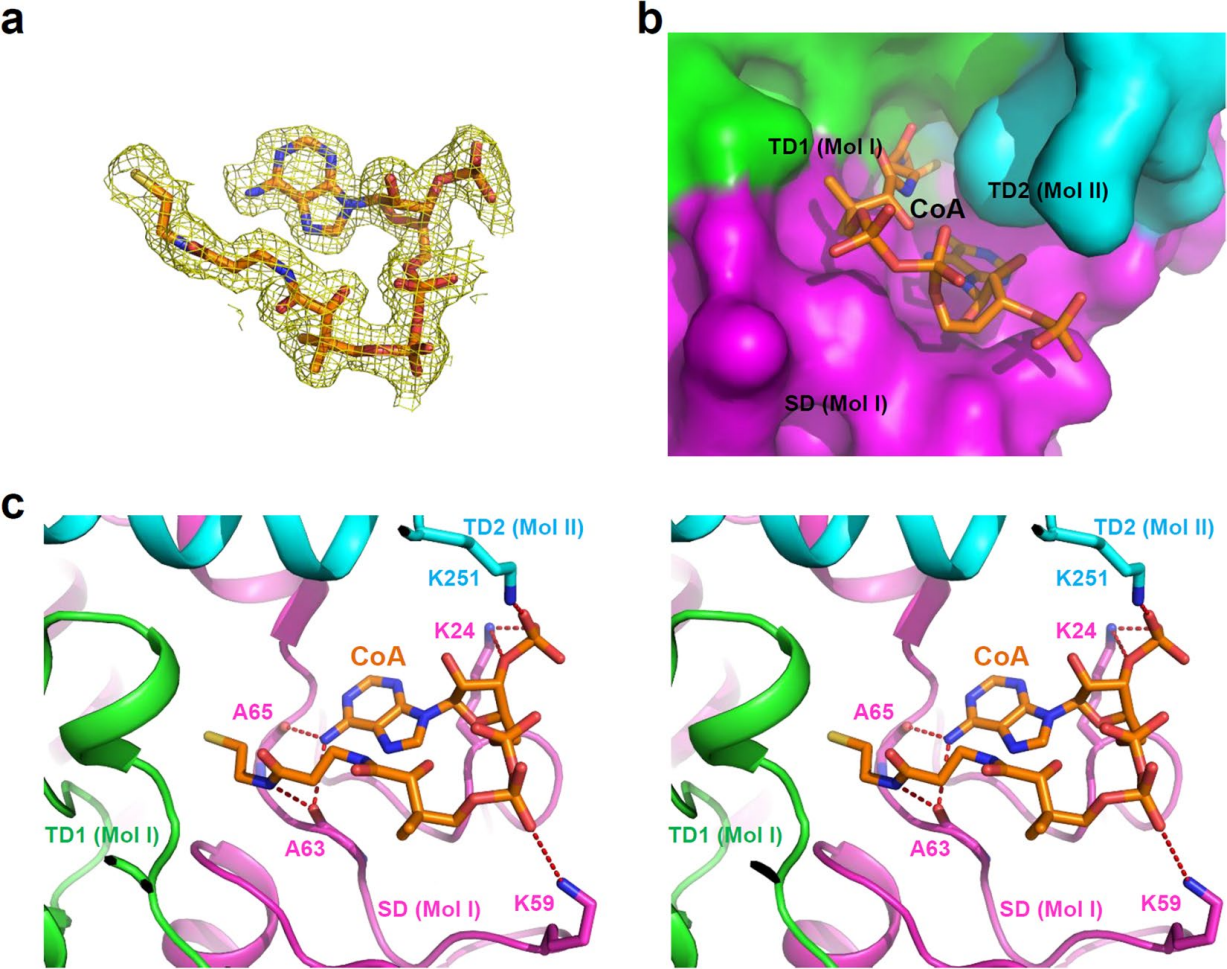
CoA binding mode of *Ms*3HPCD. (**a**) Electron density map of CoA. The Fo-Fc electron density map of the bound CoA is shown as a yellow colored mesh and contoured at 3 σ. The CoA molecule is shown as a stick model with an orange color. (**b**) A surface model of the active site of *Ms*3HPCD. The *Ms*3HPCD structure is shown as a surface model. Three domains are distinguished with different colors of magenta, green, and cyan for the SD, TD1, and TD2, respectively, and labeled appropriately. The bound CoA is presented with a stick model with an orange color. (**c**) Stereo-view of the CoA binding mode of *Ms*3HPCD. The *Ms*3HPCD structure complexed with CoA is presented with a cartoon diagram with the same color scheme as in (**b**). The residues involved in the CoA binding are shown as a stick model and labeled. The hydrogen bonds involved in the CoA binding are shown as red-colored dotted lines. The bound CoA is shown as a stick model with an orange color.

Two glutamate residues (Glu113 and Glu133), which were known to act as catalytic acid and base, respectively, are positioned at the active site of *Ms*3HPCD. In the vicinity of the catalytic residues, Ala65 and Gly110 are positioned to form an oxyanion hole during enzyme catalysis. These four residues are completely conserved among the ECH family enzymes; therefore, we suspect that *Ms*3HPCD catalyzes the enzyme reaction in a mode similar to that of other ECH enzymes^25–27^. In our current structure, the CoA molecule is tightly bound at the substrate binding site (Fig. 3a), although we did not add the molecule during the protein expression and purification procedure, which enabled us to identify the CoA binding mode of the enzyme. The CoA binding site is formed between three domains, the SD and TD1 of one molecule (Mol I), and the TD2 of the neighboring molecule (Mol II) (Fig. 3b). In the crystal structure of *Ms*3HPCD in complex with CoA, the CoA molecule is bent at the diphosphate-moiety (Fig. 3b). The pantothenic acid-moiety of CoA is tightly bound and buried in the pocket, whereas the 3′-phosphoadenosine moiety is partially exposed to the surface of *Ms*3HPCD (Fig. 3b). Three lysine residues, Lys24, Lys59, and Lys251, are located at the surface of *Ms*3HPCD and stabilize the 3′-phosphoadenosine moiety of CoA (Fig. 3c). Lys24 and Lys251 form hydrogen bonds with the phosphate moiety of the phosphoribose ring, and Lys59 stabilizes the diphosphate moiety through salt bridges (Fig. 3c). The adenosine ring is stabilized by the main chains of Ala63 and Ala65 through hydrogen bonds, and the main-chain oxygen atom of Ala63 also contributes to the stabilization of the β-cystamine moiety (Fig. 3c).

### Structural basis for 3-HP-CoA substrate specificity of *Ms*3HPCD

**Figure 4:**
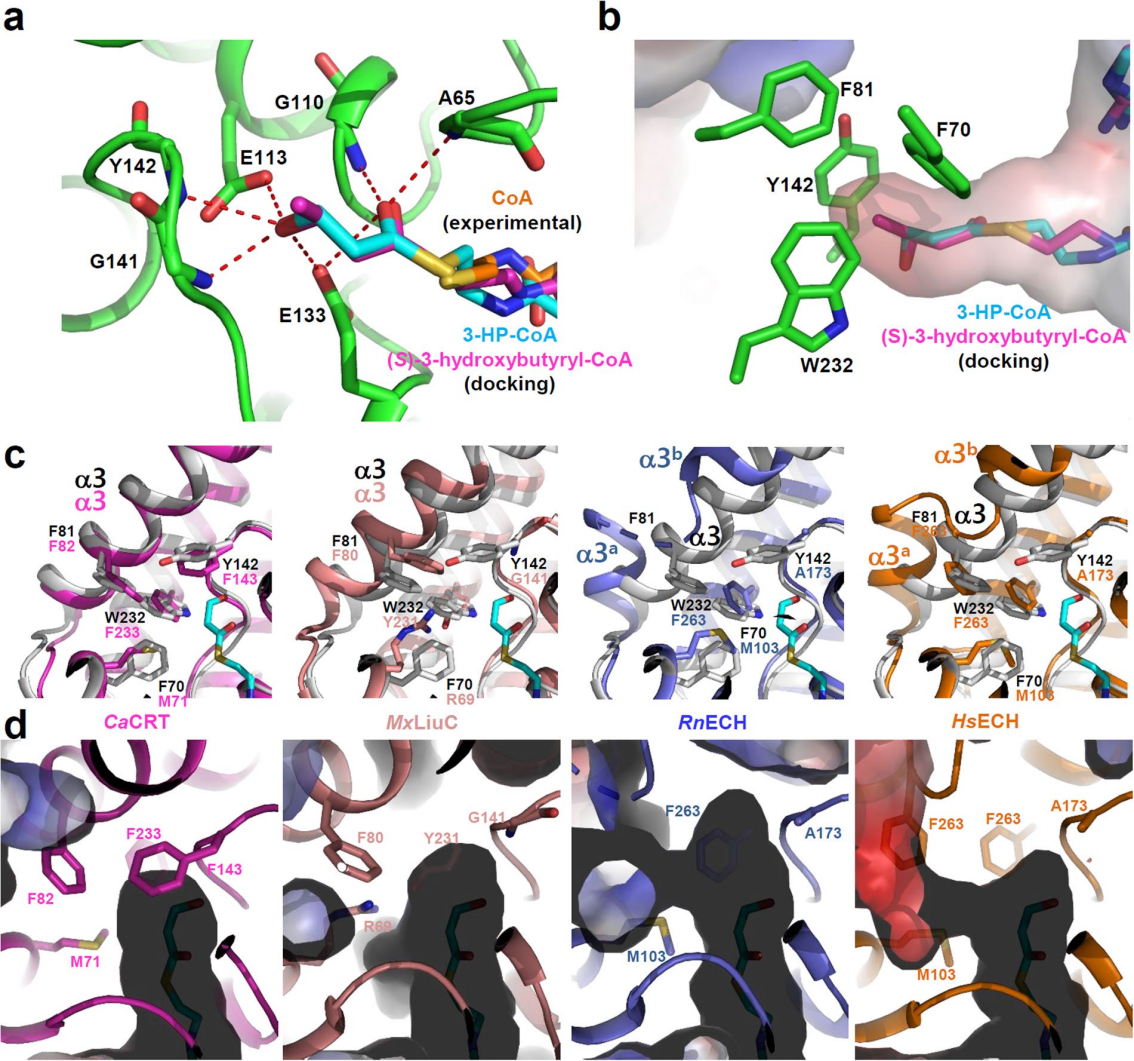
Substrate specificity of *Ms*3HPCD. (**a**) 3-HP- and (*S*)-3-hydroxybutyryl-moiety binding mode of *Ms*3HPCD. The *Ms*3HPCD structure is shown as a cartoon diagram with a green color. The residues involved in the formation of the 3-HP binding pocket are shown as stick models and labeled appropriately. The bound CoA, simulated 3-HP-CoA, and simulated (*S*)-3-hydroxybutyryl-CoA are presented with stick models with colors of cyan, orange, and magenta, respectively. Hydrogen bonds involved in the 3-HP and (*S*)-3-hydroxybutyryl-moiety binding are shown as red-colored dotted lines. (**b**) Electrostatic potential surface model of the 3-HP binding pocket of *Ms*3HPCD. The *Ms*3HPCD structure is shown as an electrostatic potential surface presentation. The simulated 3-HP-CoA and (*S*)-3-hydroxybutyryl-CoA are presented by a stick model with cyan and magenta colors. The residues involved in the formation of the 3-HP binding pocket are shown as stick models with a green color. (**c**) Structural comparison of *Ms*3HPCD with *Ca*CRT, *Mx*LiuC, *Rn*ECH, and *Hs*ECH. The structure of *Ms*3HPCD is superposed with each of those of *Ca*CRT, *Mx*LiuC, *Rn*ECH, and *Hs*ECH. The structure of *Ms*3HPCD is shown with a gray color, and those of *Ca*CRT, *Mx*LiuC, *Rn*ECH, and *Hs*ECH are with colors of magenta, salmon, light-blue, and orange, respectively. The residues involved in constitution of the enoyl-binding pocket are shown as stick models. (**d**) Electrostatic potential surface model of the 3-HP binding pocket of other ECHs. The structures of *Ca*CRT, *Mx*LiuC, *Rn*ECH, and *Hs*ECH are shown as cartoon models and electrostatic potential surface presentations with color scheme same as in (**c**). The residues involved in the formation of the enoyl-binding pocket are shown as stick models.

The ECH family enzymes utilize various types of enoyl-CoAs, with carbon chain lengths of 4 to 16 as a substrate^28^. However, because *Ms*3HPCD utilizes 3-HP-CoA, which has three carbons with a 3-hydroxyl-group, as a substrate, we expected that a relatively small binding pocket might be required for the stabilization of the 3-HP moiety. To provide structural basis for how *Ms*3HPCD accommodates 3-HP-CoA as a substrate, we performed molecular docking calculations of 3-HP-CoA into its crystal structure. Although the relative position of the CoA moiety of the simulated 3-HP-CoA is somewhat different from that of the bound CoA molecule in the complex structure, the positions of the thiol-group were almost identical between the simulated 3-HP-CoA and the bound CoA molecule (Fig. 4a), indicating that the calculated position of the 3-HP moiety might be quite similar to the actual position. The small 3-HP-binding pocket was formed deep in the enzyme and the 3-HP moiety was perfectly bound in the pocket. Four aromatic residues, such as Phe70, Phe81, Tyr142, and Trp232, play crucial roles in the formation of the unique small size pocket (Fig. 4b). Among them, two residues, Phe81 and Trp232 seem to be particularly important, because these two residues are located at the bottom of the active site and block the substrate-binding tunnel (Fig. 4b). Except for two catalytic glutamate residues, main-chains near the 3-HP moiety are mainly involved in the stabilization of polar 3-HP moiety through hydrogen bonds. The carbonyl oxygen of the 3-HP moiety forms hydrogen bonds with the main-chain nitrogen atoms of Ala65 and Gly110, and with the side-chain of Glu133 (Fig. 4a). In addition, the 3-hydroxyl-group of 3-HP moiety forms hydrogen bonds with the main-chain nitrogen atoms of Gly141 and Tyr142, and with the side-chains of Glu113 and Glu133 (Fig. 4a).

It was known that *Ms*3HPCD acts almost equally as well on (*S*)-3-hydroxybutyryl-CoA as on 3-HP-CoA^21^. To elucidate the binding mode of the (*S*)-3-hydroxybutyryl-moiety to the 3-HP binding pocket, we performed molecular docking calculations of (*S*)-3-hydroxybutyryl-CoA into its crystal structure. The (*S*)-3-hydroxybutyryl-moiety of (*S*)-3-hydroxybutyryl-CoA was perfectly matched with the 3-HP-moiety of 3-HP-CoA, and the 3-hydroxyl-group of (*S*)-3-hydroxybutyryl-moiety was stabilized in a mode identical to the 3-hydroxyl-group of 3-HP-moiety (Fig. 4a). The C4-moiety is located at the small hydrophobic pocket formed by four aromatic residues, such as Phe70, Phe81, Tyr142, and Trp232 (Fig. 4b). These observations explain how *Ms*3HPCD can accommodate both (*S*)-3-hydroxybutyryl-CoA and 3-HP-CoA as a substrate. The surface model of *Ms*3HPCD shows that four carbon length acyl-group might be a maximum size of substrate to be accommodated at the 3-HP binding pocket (Fig. 4b), indicating that the protein belongs to a short-chain enoyl-CoA hydratase. The previous study also showed that *Ms*3HPCD cannot convert (*R*)-stereoisomer of 3-hydroxybutyryl-CoA^21^, and the docking calculations of (*S*)-3-hydroxybutyryl-CoA also explain why (*R*)-3-hydroxybutyryl-CoA cannot be utilized as a substrate of *Ms*3HPCD. As we described above, the bindings of the 3-hydroxyl- and the C4-moiety into the 3-HP binding pocket are mediated through quite specific interactions (Fig. 4a). However, when (*R*)-3-hydroxybutyryl-CoA is used as a substrate, the positions of the 3-hydroxyl-group and the C4-moiety are reversed each other, resulting in improper positioning of the (*R*)-3-hydroxybutyryl-moiety in the pocket.

### Structural comparison of *Ms*3HPCD with other ECHs

To compare the 3-HP-binding pocket of *Ms*3HPCD with the enoyl-binding pocket of other ECH enzymes, we selected four ECH enzymes with different substrate specificities, including ECH from *Homo sapiens* (*Hs*ECH, PDB code 2HW5), ECH from *Rattus norvegicus* (*Rn*ECH, PDB code 1MJ3)^22^, crotonase from *Clostridium acetobutylicum* (*Ca*CRT, PDB code 5Z7R)^29^, and 3-Hydroxy-3-Methylglutaconyl-CoA Dehydratase from *Myxococcus xanthus* (*Mx*LiuC, PDB code 5JBX)^30^. *Rn*ECH and *Hs*ECH are known to utilize long chain enoyl-CoA substrates, whereas *Ca*CRT and *Mx*LiuC accommodate short chain enoyl-CoA substrates. When we superimposed the structure of *Ms*3HPCD on those of *Hs*ECH, *Rn*ECH, *Ca*CRT, and *Mx*LiuC, the overall structures were quite similar to each other, with root-mean-square deviation (R.M.S.D.) values of 0.912, 0.823, 0.662, and 1.134 Å over 217, 212, 233, and 198 atoms, respectively. However, remarkable structural differences were observed at the bottom of the 3-HP-binding pocket, especially for the conformation of the α3 helix and the residues constituting the 3-HP-binding pocket (Fig. 4c). *Ms*3HPCD and *Ca*CRT have a tightly-formed α3 helix at almost identical position, and the residues constituting the 3-HP-binding pocket were quite similar to each other. *Ca*CRT has Met71, Phe143, and Phe233 at the positions corresponding to Phe70, Tyr142, and Trp232 in *Ms*3HPCD (Fig. 4c). *Ca*CRT is known to utilize (*S*)-3-hydroxybutyryl-CoA as a substrate, and the observations further support how *Ms*3HPCD can accommodate both (*S*)-3-hydroxybutyryl-CoA and 3-HP-CoA as a substrate. *Mx*LiuC also has a rigid α3 helix, however, the helix is located slightly further away from the active site compared with that of *Ms*3HPCD (Fig. 4c). Moreover, the residues constituting the 3-HP-binding pocket were also quite different. In *Mx*LiuC, the positions corresponding to Phe70, Tyr142, and Trp232 in *Ms*3HPCD, are Arg69, Gly141, and Tyr231, making the 3-Hydroxy-3-Methylglutaconyl-binding pocket for *Mx*LiuC larger than the 3-HP-binding pocket of *Ms*3HPCD (Fig. 4d). Importantly, Arg69 might provide hydrophilicity to stabilize the somewhat hydrophilic substrate. When we compared *Rn*ECH and *Hs*ECH with *Ms*3HPCD, we observed that these enzymes exhibit the highly flexible helix-loop-helix conformation at the corresponding region of the α3 helix in *Ms*3HPCD (Fig. 4c). The helix-loop-helix in *Hs*ECH and *Rn*ECH, especially the first helix, protrudes out from the substrate binding site, which results in the formation of a long substrate binding pocket to accommodate the long chain enoyl-CoA as a substrate (Fig. 4d). These structural observations indicated that the conformation of the α3-helix and the residues constituting the enoyl-binding pocket are the key elements that determine the substrate specificity of the ECH family enzymes.

### Phylogenetic tree analysis of ECH enzymes

**Figure 5:**
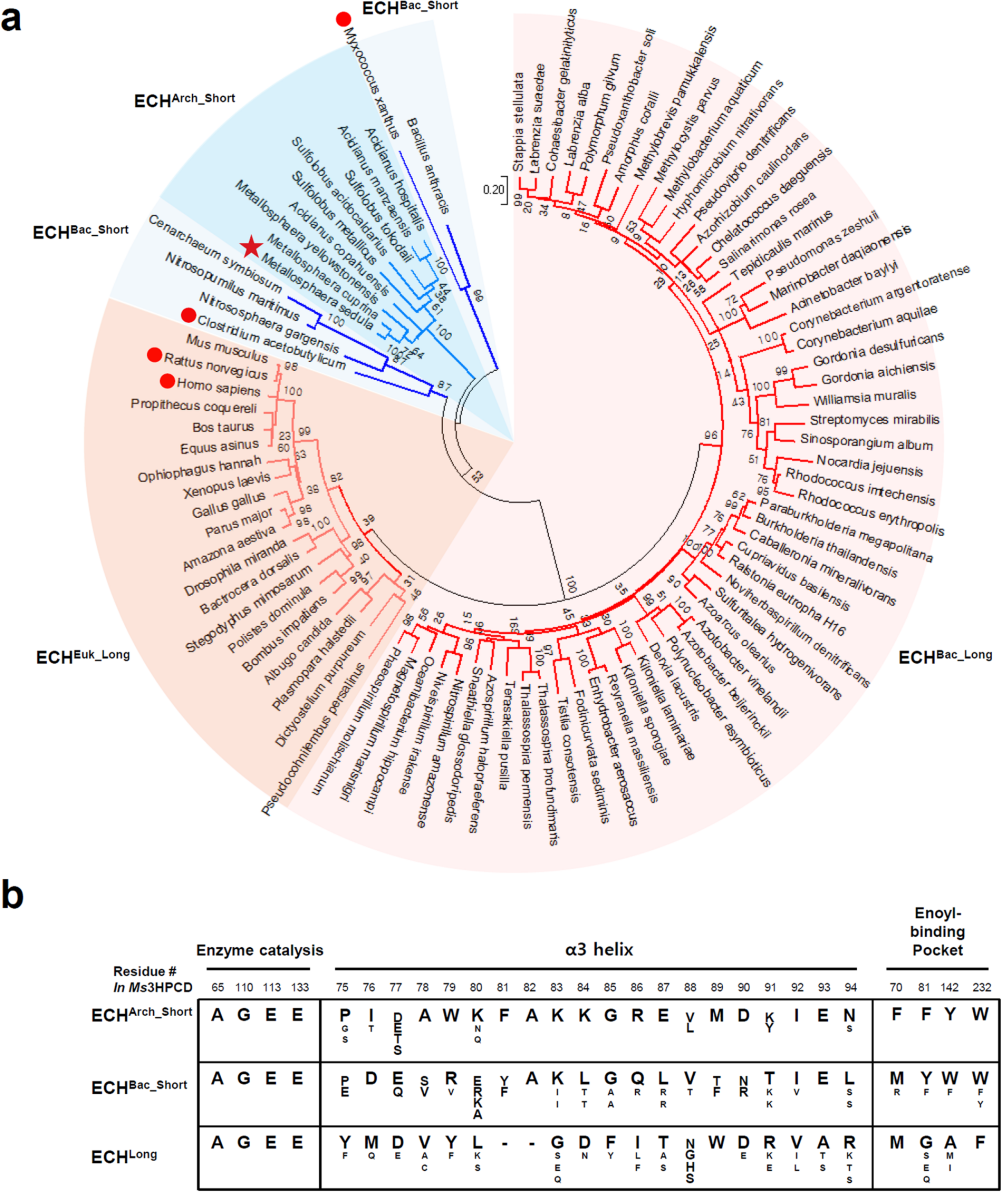
Phylogenetic analysis of ECH enzymes. (**a**) Unrooted Maximum Likelihood tree of ECH enzymes. The phylogenetic tree is drawn as a circle model. Bootstrap values are shown at each node as percentage of 100 replicates. ECH subgroups are labeled and distinguished with different colors. *Ms*3HPCD is indicated by a star mark and the structures used for the comparison with *Ms*3HPCD are indicated by red colored dots. (**b**) Amino-acid sequence alignment of key residues in ECH enzymes. The key residues involved in the enzyme catalysis, α3 helix, and enoyl-binding pocket in ECHs are aligned.

Our study revealed that substrate specificities of the ECH family enzymes are determined by the conformation of the α3 helix and the residues constituting the enoyl-CoA binding pocket. The ECH enzymes such as *Rn*ECH and *Hs*ECH exhibit a highly flexible helix-loop-helix conformation at the corresponding region of the α3 helix in *Ms*3HPCD to accommodate long-chain enoyl-CoAs as a substrate (Fig. 4c). However, ECH enzymes such as *Ca*CRT and *Mx*LiuC have a tightly-formed α3 helix to accommodate short-chain enoyl-CoAs as a substrate (Fig. 4c). We then selected 92 ECH family enzymes from phylogenetically diverse organisms and performed a maximum-likelihood phylogenetic tree analysis. The ECH enzymes could be largely divided into two types, ECH^Long^ and ECH^Short^ (Fig. 5a). Among the 92 ECH enzymes, 77 enzymes, including *Rn*ECH and *Hs*ECH, belong to ECH^Long^, and they might have a substrate specificity for long-chain enoyl-CoAs (Fig. 5a). The ECH^Long^ enzymes can be further classified into two sub-types, bacterial (ECH^Bac_Long^) and eukaryotic (ECH^Euk_Long^), according to their origin. The remaining 15 ECH enzymes, including *Ms*3HPCD, *Ca*CRT and *Mx*LiuC, belong to ECH^Short^ and they might have a substrate specificity for short-chain enoyl-CoAs. The ECH^Short^ enzymes can be further classified into two sub-types (Fig. 5a), bacterial (ECH^Bac_Short^) and archaeal (ECH^Arc_Short^), according to their origin. *Ca*CRT and *Mx*LiuC belong to ECH^Bac_Short^ and *Ms*3HPCD belongs to ECH^Arc_Short^.

We also compared the key amino acids involved in enzyme catalysis and substrate binding among the ECH family enzymes. First, we compared the amino acids involved in enzyme catalysis and formation of the oxyanion hole, and all four amino acids, Ala65, Gly110, Glu113, and Glu133 in *Ms*3HPCD, were completely conserved among all ECH enzymes (Fig. 5b). These observations indicate that ECH enzymes catalyze the enzyme reaction in an identical mode. Next, we compared the amino acids constituting the α3 helix. Surprisingly, compared with the ECH^Short^ enzymes, all ECH^Long^ enzymes showed deletion of two amino acids at the α3 helix (Fig. 5b). We suspected that the amino acid deletion in the ECH^Long^ enzymes prevent the α3 helix from having a tight conformation, instead of forming the helix-loop-helix structure, which enables the enzymes to accommodate long-chain enoyl-CoAs as a substrate. In addition, amino acids composing the α3 helix were quite different between ECH^Bac_Short^ and ECH^Arc_Short^, but were highly homologous within each sub-type (Fig. 5b). Finally, we compared the amino acids constituting the enoyl-binding pocket. Interestingly, all four aromatic amino acids, i.e. Phe70, Phe81, Tyr142, and Trp232 in *Ms*3HPCD, were completely conserved among the ECH^Arc_Short^ enzymes (Fig. 5b). These residues play a crucial role in constituting a small 3-HP-binding pocket in *Ms*3HPCD. The amino acids constituting the enoyl-binding pocket in the ECH^Bac_Short^ enzymes are somewhat different from those of the ECH^Arc_Short^ enzymes, and it seems that the amino acids forming the enoyl-binding pocket play an important role in distinguishing these two sub-groups.

In summary, we report the first crystal structure of *Ms*3HPCD in complex with CoA, and provide a structural insight into how *Ms*3HPCD utilizes both (*S*)-3-hydroxybutyryl-CoA and 3-HP-CoA as a substrate. Moreover, we suggest that the conformation of the α3 helix and the residues constituting the enoyl-binding pocket are the key structural elements that determine the substrate specificity of the ECH family enzymes. The phylogenetic tree analysis of the ECH enzymes also suggest that the ECH enzymes from the phylum *Crenarchaeota* have highly conserved amino acids that determine the substrate specificity of the enzyme.

## Methods

### Preparation of *Ms*3HPCD proteins

The *Ms*3HPCD coding gene (Met1-Lys256, M.W. 28.3 kDa) was amplified from the *Metallosphaera sedula* chromosome as the template through a polymerase chain reaction (PCR). Using the NdeI and XhoI restriction enzymes, the PCR products were then sub-cloned into pET30a (Novagen) with 6x-His tag at the C-terminus. The resulting expression vector pET30a: *Ms*3HPCD was transformed into an *Escherichia coli* BL21 (DE3)-T1^R^ strain, which was grown in 1 L of LB medium containing kanamycin (50 mgL^−1^) at 37 °C to OD600 of 0.6. The *Ms*3HPCD protein expression was induced by adding 1.0 mM Isopropyl 1-thio-β-D-galactopyranoside (IPTG). After 20 h at 18 °C, the cells were harvested by centrifugation at 4000 rpm for 20 minute. The cell pellet was resuspended in buffer A, containing 40 mM Tris-HCl pH 8.0, and disrupted by ultrasonication. The cell debris was removed through centrifugation at 13,500 × g for 25 min, and the lysate was loaded onto a Ni-NTA agarose column (QIAGEN). After washing with buffer A containing 25 mM imidazole, the bound proteins were eluted with 300 mM imidazole in buffer A. Finally, the trace amount of contaminants was removed by a size exclusion chromatography using a HiPrep 26/60 Sephacryl S-300 HR column (GE Healthcare Life Sciences) equilibrated with buffer A. All purification experiments were performed at 4 °C. SDS-polyacrylamide gel electrophoresis analysis of the purified proteins showed a single polypeptide of 28.3 kDa that corresponded to the estimated molecular weight of the *Ms*3HPCD monomer. The purified protein was concentrated to 41 mg mL^−1^ in 40 mM Tris-HCl, pH 8.0.

### Crystallization of *Ms*3HPCD

Crystallization of the purified *Ms*3HPCD protein was initially performed with commercially available sparse-matrix screens including Index, PEG ion I and II (Hampton Research), Wizard Classic I and II, Wizard CRYO I and II (Rigaku Reagents) and Structure Screen I and II (Molecular Dimensions), using the hanging-drop vapor-diffusion method at 20 °C. Each experiment consisted of mixing 1.0 µL protein solution (41 mg mL^−1^ in 40 mM Tris-HCl, pH8.0) with 1.0 µL of reservoir solution and equilibrating the drop against 50 µL reservoir solution. *Ms*3HPCD crystals were observed from several crystallization screening conditions. After several optimization steps for crystal improvement, crystals of the best quality appeared in 4 days using a reservoir solution consisting of 10% (w/v) polyethylene glycol (PEG) 8000, 0.1 M Sodium-potassium phosphate, pH 6.2, 0.2 M Sodium Chloride, and 10 mM Ethylenediaminetetraacetic acid (EDTA) disodium salt dehydrate at 20 °C.

### Data collection and structure determination of *Ms*3HPCD

The crystals were transferred to a cryo-protectant solution composed of 10% (w/v) polyethylene glycol (PEG) 8000, 0.1 M Sodium-potassium phosphate, pH 6.2, 0.2 M Sodium Chloride, 10 mM Ethylenediaminetetraacetic acid (EDTA) disodium salt dehydrate and 30% (v/v) glycerol, fished out with a loop larger than the crystals, and flash-frozen by immersion in liquid nitrogen. Data was collected to a resolution of 1.8 Å at 7 A beamline of the Pohang Accelerator Laboratory (PAL, Pohang, Korea), using a Quantum 270 CCD detector (ADSC, USA)^31^. All data were indexed, integrated, and scaled together using the HKL2000 software package^32^. The crystals of *Ms*3HPCD in complex with CoA belonged to *P*21 with unit cell parameters *a* = 84.685 Å, *b* = 130.17 Å, *c* = 84.791 Å, α = γ = 90°, β = 119.57°. Assuming six *Ms*3HPCD molecules in asymmetric unit, the crystal volume per unit of protein mass was 2.39 Å^3^ Da^−1^, which means the solvent content was approximately 48.65%^33^. The structure of *Ms*3HPCD in complex with CoA was determined by molecular replacement with the CCP4 version of MOLREP^34^ using the structure of enoyl-CoA hydratase from *Mycobacterium tuberculosis* (*Mt*ECH, PDB code 3PZK) as a search model. Model building was performed manually using the program WinCoot^35^, and refinement was performed with CCP4 refmac5^36^. The data statistics are summarized in Table 1 and the refined *Ms*3HPCD structure in complex with CoA was deposited in the protein data bank under the PDB code 5ZAI.

### Size-exclusion chromatographic analysis

To investigate the oligomerization of *Ms*3HPCD, analytical size-exclusion chromatography was performed using a Superdex increase 200 10/300 GL column (GE Healthcare Life Sciences) equilibrated with 40 mM Tris-HCl, pH 8.0 and 150 mM NaCl. Protein sample of 1 mL with concentration of 1 mg mL^−1^ was analyzed. The molecular weights of the eluted samples were calculated based on the calibration curve drawn using standard samples of ferritin (440 kDa), conalbumin (75 kDa), carbonic anhydrase (29 kDa), and ribonuclease A (13.7 kDa) (GE Healthcare Life Sciences).

### Molecular docking simulation of *Ms*3HPCD

Molecular docking simulations of 3-HP-CoA and (*S*)-3-hydroxybutyryl-CoA to *Ms*3HPCD structure were performed by AutoDock Vina software^37^. The ligand molecule of *Ms*3HPCD, 3-HP-CoA and (*S*)-3-hydroxybutyryl-CoA, were prepared using the JLigand software. For the docking simulation, the pdbqt files were generated using AutoDock Vina manual. The grid size for 3-HP-CoA was x = 16, y = 22, z = 16, and grid box was centered at x = 16.811, y = −8.113, z = 3.327. The final conformations produced in this simulation were checked using PyMOL software^38^.

### Phylogenetic tree analysis

Iterative searching for 3HPCD-like proteins was performed by Basic Local Alignment Search Tool (BLAST) in National Center for Biotechnology information (NCBI) server using position-specific iterated BLAST (PSI-BLAST) method^39^. Multiple alignment was performed by Clustal omega^40^. The evolutionary history was inferred by using the Maximum Likelihood method based on the Le_Gascuel_2008 model^41^. The tree with the highest log likelihood (−20025.9646) is shown. Initial tree(s) for the heuristic search were obtained automatically by applying Neighbor-Join and BioNJ algorithms to a matrix of pairwise distances estimated using a JTT model, and then selecting the topology with superior log likelihood value. A discrete Gamma distribution was used to model evolutionary rate differences among sites (5 categories (+*G*, parameter = 0.9794)). The rate variation model allowed for some sites to be evolutionarily invariable ([+*I*], 9.9777% sites). The tree is drawn to scale, with branch lengths measured in the number of substitutions per site. The analysis involved 92 amino acid sequences. All positions with less than 95% site coverage were eliminated. That is, fewer than 5% alignment gaps, missing data, and ambiguous amino acids were allowed at any position. There were a total of 254 positions in the final dataset. Evolutionary analyses were conducted in MEGA7^42^.

## Electronic supplementary material

Validation report for structure
