# Supplementary material for: Structural Insight into Substrate Specificity of 3-Hydroxypropionyl-Coenzyme A Dehydratase from Metallosphaera sedula
Source: Sci Rep. 2018 Jul 16;8:10692. doi: 10.1038/s41598-018-29070-w (PMC6048173; doi:10.1038/s41598-018-29070-w)
Supplement: Supplementary file 1 — Validation report for structure [file 41598_2018_29070_MOESM1_ESM.pdf]

WORLDWIDE PDB PROTEIN DATA BANK

Preliminary Full wwPDB X-ray Structure Validation Report (i)

May 17, 2018 – 09:33 PM EDT

This is a Preliminary Full wwPDB X-ray Structure Validation Report.

This report is produced by the standalone wwPDB validation server.
The structure in question has not been deposited to the wwPDB.
This report should not be submitted to journals.

We welcome your comments at *validation@mail.wwpdb.org*
A user guide is available at
https://www.wwpdb.org/validation/2017/XrayValidationReportHelp
with specific help available everywhere you see the (i) symbol.

PRELIMINARY VALIDATION REPORT

The following versions of software and data (see references (i)) were used in the production of this report:

MolProbity	:	4.02b-467
Mogul	:	1.7.3 (157068), CSD as539be (2018)
Xtriage (Phenix)	:	1.13
EDS	:	rb-20031021
Percentile statistics	:	20171227.v01 (using entries in the PDB archive December 27th 2017)
Refmac	:	5.8.0158
CCP4	:	7.0 (Gargrove)
Ideal geometry (proteins)	:	Engh & Huber (2001)
Ideal geometry (DNA, RNA)	:	Parkinson et al. (1996)
Validation Pipeline (wwPDB-VP)	:	rb-20031021

1 Overall quality at a glance (i)

The following experimental techniques were used to determine the structure:
X-RAY DIFFRACTION

The reported resolution of this entry is 1.80 Å.

Percentile scores (ranging between 0-100) for global validation metrics of the entry are shown in the following graphic. The table shows the number of entries on which the scores are based.

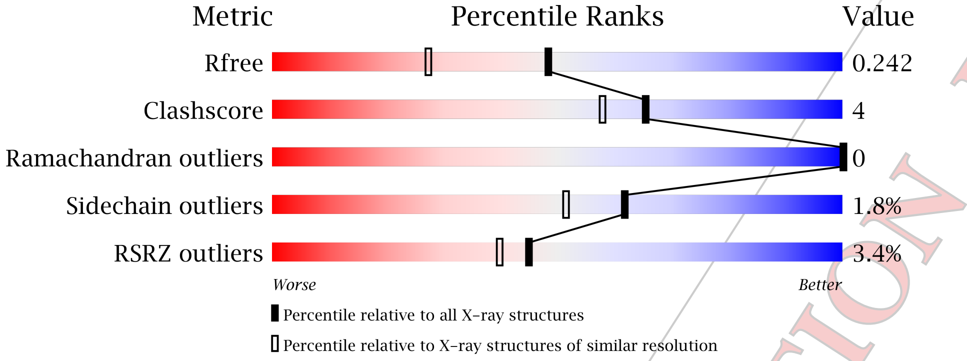

Metric	Whole archive (#Entries)	Similar resolution (#Entries, resolution range(Å))
R_{free}	111664	5253 (1.80-1.80)
Clashscore	122126	6077 (1.80-1.80)
Ramachandran outliers	120053	6011 (1.80-1.80)
Sidechain outliers	120020	6010 (1.80-1.80)
RSRZ outliers	108989	5157 (1.80-1.80)

The table below summarises the geometric issues observed across the polymeric chains and their fit to the electron density. The red, orange, yellow and green segments on the lower bar indicate the fraction of residues that contain outliers for >=3, 2, 1 and 0 types of geometric quality criteria. A grey segment represents the fraction of residues that are not modelled. The numeric value for each fraction is indicated below the corresponding segment, with a dot representing fractions <=5% The upper red bar (where present) indicates the fraction of residues that have poor fit to the electron density. The numeric value is given above the bar.

Mol	Chain	Length	Quality of chain
1	A	259	2% / 90% 9% •
1	B	259	3% / 88% 10% •
1	C	259	3% / 88% 12%
1	D	259	3% / 90% 9%
1	E	259	2% / 90% 9% •

Continued on next page...

PRELIMINARY VALIDATION REPORT

Continued from previous page...

Mol	Chain	Length	Quality of chain
1	F	259	6% 90% 9% •

PRELIMINARY VALIDATION REPORT

2 Entry composition (i)

There are 5 unique types of molecules in this entry. The entry contains 13087 atoms, of which 0 are hydrogens and 0 are deuteriums.

In the tables below, the ZeroOcc column contains the number of atoms modelled with zero occupancy, the AltConf column contains the number of residues with at least one atom in alternate conformation and the Trace column contains the number of residues modelled with at most 2 atoms.

- Molecule 1 is a protein called 3-Hydroxypropionyl-CoA dehydratase.

Mol	Chain	Residues	Atoms	ZeroOcc	AltConf	Trace
1	A	257	Total C N O S<br>1986 1262 342 374 8	0	1	0
1	B	257	Total C N O S<br>2008 1274 350 376 8	0	3	0
1	C	259	Total C N O S<br>1996 1268 343 377 8	0	1	0
1	D	258	Total C N O S<br>1990 1264 343 375 8	0	1	0
1	E	257	Total C N O S<br>1986 1262 342 374 8	0	1	0
1	F	257	Total C N O S<br>1975 1256 338 373 8	0	0	0

- Molecule 2 is COENZYME A (three-letter code: COA) (formula: $C_{21}H_{36}N_7O_{16}P_3S$).

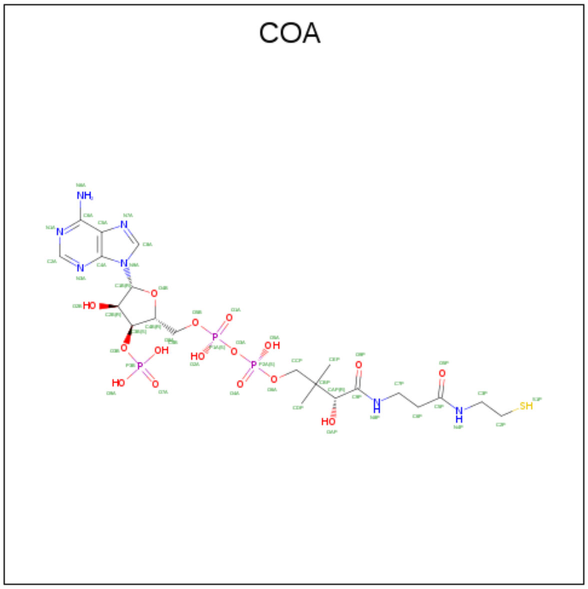

Mol	Chain	Residues	Atoms	ZeroOcc	AltConf
2	G	1	Total C N O P S<br>48 21 7 16 3 1	0	0
2	G	1	Total C N O P S<br>48 21 7 16 3 1	0	0
2	G	1	Total C N O P S<br>48 21 7 16 3 1	0	0
2	G	1	Total C N O P S<br>48 21 7 16 3 1	0	0

- Molecule 3 is 1,2-ETHANEDIOL (three-letter code: EDO) (formula: $C_2H_6O_2$).

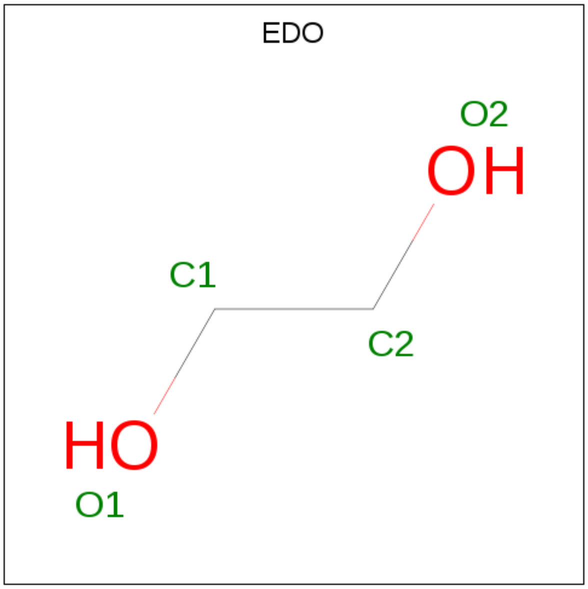

Mol	Chain	Residues	Atoms	ZeroOcc	AltConf
3	H	1	Total C O<br>4 2 2	0	0
3	H	1	Total C O<br>4 2 2	0	0
3	H	1	Total C O<br>4 2 2	0	0
3	H	1	Total C O<br>4 2 2	0	0
3	H	1	Total C O<br>4 2 2	0	0
3	H	1	Total C O<br>4 2 2	0	0
3	H	1	Total C O<br>4 2 2	0	0
3	H	1	Total C O<br>4 2 2	0	0

Continued on next page...

PRELIMINARY VALIDATION REPORT

Continued from previous page...

Mol	Chain	Residues	Atoms	ZeroOcc	AltConf
3	H	1	Total C O<br>4 2 2	0	0
3	H	1	Total C O<br>4 2 2	0	0
3	H	1	Total C O<br>4 2 2	0	0

- Molecule 4 is GLYCEROL (three-letter code: GOL) (formula: $C_3H_8O_3$).

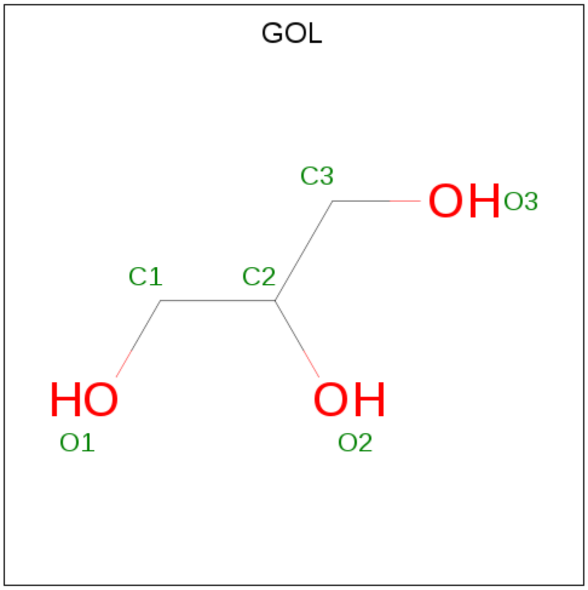

Mol	Chain	Residues	Atoms	ZeroOcc	AltConf
4	I	1	Total C O<br>6 3 3	0	0
4	I	1	Total C O<br>6 3 3	0	0
4	I	1	Total C O<br>6 3 3	0	0
4	I	1	Total C O<br>6 3 3	0	0
4	I	1	Total C O<br>6 3 3	0	0
4	I	1	Total C O<br>6 3 3	0	0
4	I	1	Total C O<br>6 3 3	0	0
4	I	1	Total C O<br>6 3 3	0	0

Continued on next page...

PRELIMINARY VALIDATION REPORT

Continued from previous page...

Mol	Chain	Residues	Atoms	ZeroOcc	AltConf
4	I	1	Total C O<br>6 3 3	0	0
4	I	1	Total C O<br>6 3 3	0	0

- Molecule 5 is water.

Mol	Chain	Residues	Atoms	ZeroOcc	AltConf
5	K	850	Total O<br>850 850	0	0

PRELIMINARY VALIDATION REPORT

3 Residue-property plots ⓘ

These plots are drawn for all protein, RNA and DNA chains in the entry. The first graphic for a chain summarises the proportions of the various outlier classes displayed in the second graphic. The second graphic shows the sequence view annotated by issues in geometryand electron density. Residues are color-coded according to the number of geometric quality criteria for which they contain at least one outlier: green = 0, yellow = 1, orange = 2 and red = 3 or more. A red dot above a residue indicates a poor fit to the electron density (RSRZ > 2). Stretches of 2 or more consecutive residues without any outlier are shown as a green connector. Residues present in the sample, but not in the model, are shown in grey.

• Molecule 1: 3-Hydroxypropionyl-CoA dehydratase

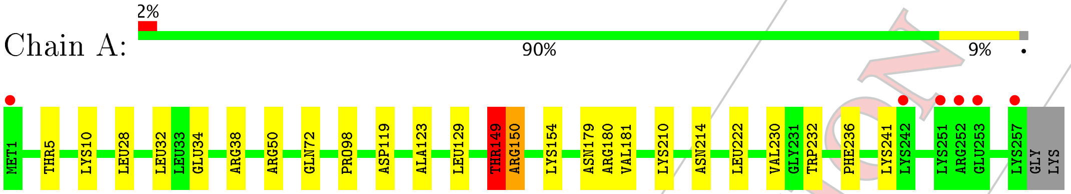

• Molecule 1: 3-Hydroxypropionyl-CoA dehydratase

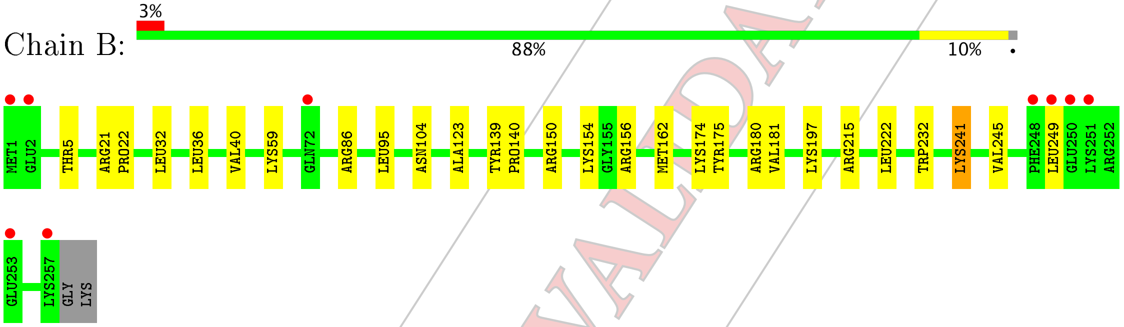

• Molecule 1: 3-Hydroxypropionyl-CoA dehydratase

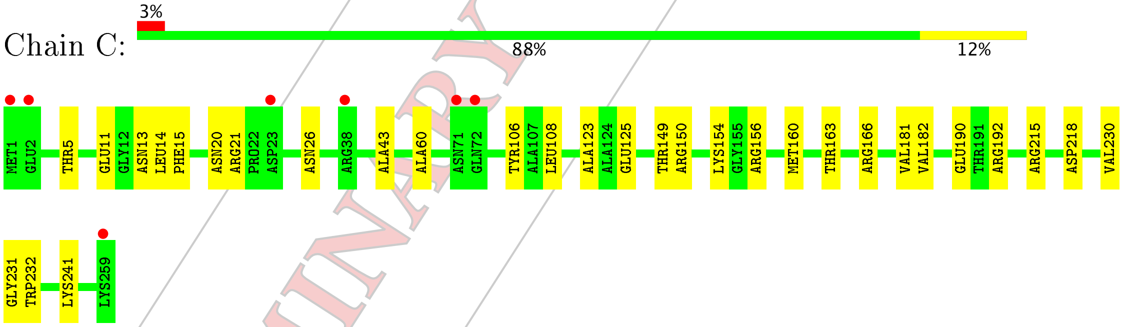

• Molecule 1: 3-Hydroxypropionyl-CoA dehydratase

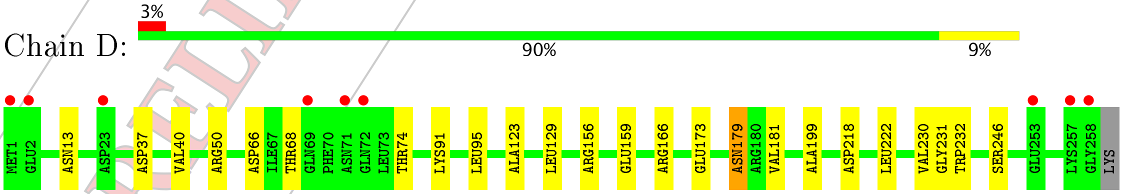

PRELIMINARY VALIDATION REPORT

- Molecule 1: 3-Hydroxypropionyl-CoA dehydratase

Chain E:

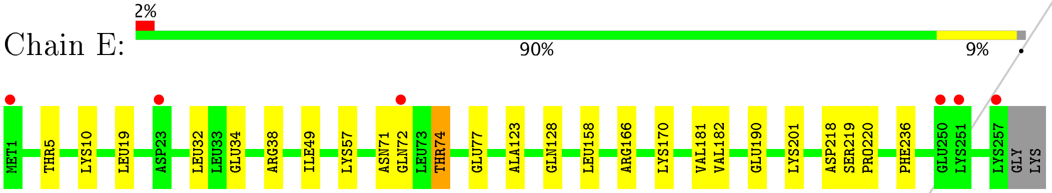

- Molecule 1: 3-Hydroxypropionyl-CoA dehydratase

Chain F:

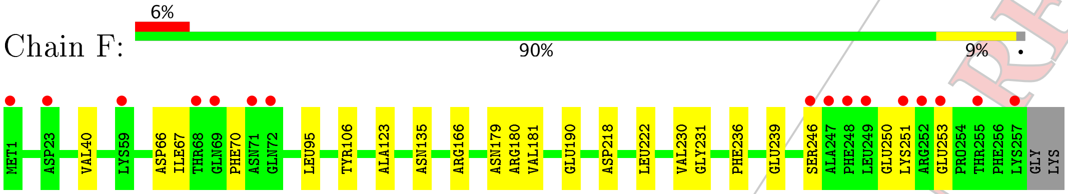

PRELIMINARY VALIDATION REPORT

4 Data and refinement statistics (i)

Property	Value	Source
Space group	P 1 21 1	Depositor
Cell constants<br>a, b, c, α, β, γ	84.69Å 130.17Å 84.79Å<br>90.00° 119.57° 90.00°	Depositor
Resolution (Å)	25.83 – 1.80<br>25.81 – 1.80	Depositor<br>EDS
% Data completeness<br>(in resolution range)	98.0 (25.83-1.80)<br>98.1 (25.81-1.80)	Depositor<br>EDS
R_{merge}	(Not available)	Depositor
R_{sym}	(Not available)	Depositor
$< I/\sigma(I) >$ [1]	1.38 (at 1.79Å)	Xtriage
Refinement program	REFMAC 5.8.0222	Depositor
R, R_{free}	0.183 , 0.229<br>0.197 , 0.242	Depositor<br>DCC
R_{free} test set	7078 reflections (4.87%)	wwPDB-VP
Wilson B-factor (Å^2)	17.4	Xtriage
Anisotropy	0.229	Xtriage
Bulk solvent k_{sol}(e/Å^3), B_{sol}(Å^2)	0.39 , 50.1	EDS
L-test for twinning[2]	$< \|L\| > = 0.49$, $< L^2 > = 0.32$	Xtriage
Estimated twinning fraction	0.000 for -h-l,k,h<br>0.000 for l,k,-h-l<br>0.016 for h,-k,-h-l<br>0.017 for -h-l,-k,l<br>0.019 for l,-k,h	Xtriage
F_o,F_c correlation	0.95	EDS
Total number of atoms	13087	wwPDB-VP
Average B, all atoms (Å^2)	27.0	wwPDB-VP

Xtriage's analysis on translational NCS is as follows: *The largest off-origin peak in the Patterson function is 3.51% of the height of the origin peak. No significant pseudotranslation is detected.*

[1] Intensities estimated from amplitudes.

[2] Theoretical values of $< |L| >$, $< L^2 >$ for acentric reflections are 0.5, 0.333 respectively for untwinned datasets, and 0.375, 0.2 for perfectly twinned datasets.

PRELIMINARY VALIDATION REPORT

5 Model quality (i)

5.1 Standard geometry (i)

Bond lengths and bond angles in the following residue types are not validated in this section: COA, GOL, EDO

The Z score for a bond length (or angle) is the number of standard deviations the observed value is removed from the expected value. A bond length (or angle) with $|Z| > 5$ is considered an outlier worth inspection. RMSZ is the root-mean-square of all Z scores of the bond lengths (or angles).

Mol	Chain	Bond lengths		Bond angles	
		RMSZ	$\#\|Z\| > 5$	RMSZ	$\#\|Z\| > 5$
1	A	0.64	0/2014	0.74	1/2710 (0.0%)
1	B	0.60	0/2036	0.75	1/2738 (0.0%)
1	C	0.56	0/2024	0.72	3/2723 (0.1%)
1	D	0.66	0/2018	0.75	0/2715
1	E	0.57	0/2014	0.72	0/2710
1	F	0.59	0/2003	0.71	0/2696
All	All	0.61	0/12109	0.73	5/16292 (0.0%)

Chiral center outliers are detected by calculating the chiral volume of a chiral center and verifying if the center is modelled as a planar moiety or with the opposite hand.A planarity outlier is detected by checking planarity of atoms in a peptide group, atoms in a mainchain group or atoms of a sidechain that are expected to be planar.

Mol	Chain	#Chirality outliers	#Planarity outliers
1	A	0	4
1	B	0	2
All	All	0	6

There are no bond length outliers.

All (5) bond angle outliers are listed below:

Mol	Chain	Res	Type	Atoms	Z	Observed(^{o})	Ideal(^{o})
1	C	150	ARG	NE-CZ-NH2	-6.42	117.09	120.30
1	C	156	ARG	NE-CZ-NH2	6.06	123.33	120.30
1	C	156	ARG	NE-CZ-NH1	-5.74	117.43	120.30
1	B	86	ARG	NE-CZ-NH2	-5.22	117.69	120.30
1	A	149	THR	O-C-N	-5.14	114.47	122.70

There are no chirality outliers.

All (6) planarity outliers are listed below:

PRELIMINARY VALIDATION REPORT

Mol	Chain	Res	Type	Group
1	A	149	THR	Mainchain
1	A	150[A]	ARG	Sidechain
1	A	180	ARG	Sidechain
1	B	180	ARG	Sidechain
1	B	21	ARG	Sidechain

5.2 Too-close contacts (i)

In the following table, the Non-H and H(model) columns list the number of non-hydrogen atoms and hydrogen atoms in the chain respectively. The H(added) column lists the number of hydrogen atoms added and optimized by MolProbity. The Clashes column lists the number of clashes within the asymmetric unit, whereas Symm-Clashes lists symmetry related clashes.

Mol	Chain	Non-H	H(model)	H(added)	Clashes	Symm-Clashes
1	A	1986	0	2066	19	0
1	B	2008	0	2090	19	0
1	C	1996	0	2075	27	0
1	D	1990	0	2069	20	0
1	E	1986	0	2066	19	0
1	F	1975	0	2054	16	0
2	G	192	0	128	7	0
3	H	44	0	66	7	0
4	I	60	0	80	2	0
5	K	850	0	0	23	0
All	All	13087	0	12694	110	0

The all-atom clashscore is defined as the number of clashes found per 1000 atoms (including hydrogen atoms). The all-atom clashscore for this structure is 4.

All (110) close contacts within the same asymmetric unit are listed below, sorted by their clash magnitude.

Atom-1	Atom-2	Interatomic distance (Å)	Clash overlap (Å)
1:C:166:ARG:HH22	2:G:4:COA:CEP	1.91	0.84
1:E:74:THR:HG21	5:K:784:HOH:O	1.79	0.81
1:D:179:ASN:ND2	5:K:803:HOH:O	2.15	0.80
1:A:230:VAL:HG12	1:F:230:VAL:HG12	1.64	0.79
1:F:246:SER:O	1:F:250:GLU:HG2	1.84	0.78
1:D:74:THR:HG22	5:K:190:HOH:O	1.85	0.77
1:F:40:VAL:HG13	1:F:95:LEU:HD11	1.66	0.77
1:D:74:THR:CG2	5:K:190:HOH:O	2.39	0.69
1:A:214:ASN:HD21	1:B:154:LYS:NZ	1.91	0.69

Continued on next page...

PRELIMINARY VALIDATION REPORT

Continued from previous page...

Atom-1	Atom-2	Interatomic distance (Å)	Clash overlap (Å)
1:C:166:ARG:HH22	2:G:4:COA:H143	1.58	0.68
1:C:125:GLU:O	5:K:237:HOH:O	2.13	0.65
1:E:74:THR:HG23	5:K:130:HOH:O	1.98	0.62
1:A:214:ASN:HD21	1:B:154:LYS:HZ2	1.49	0.60
1:F:179:ASN:ND2	5:K:821:HOH:O	2.34	0.60
1:D:173:GLU:OE2	5:K:647:HOH:O	2.17	0.60
1:D:173:GLU:OE1	1:D:181:VAL:HG22	2.02	0.59
1:B:174:LYS:NZ	5:K:573:HOH:O	2.35	0.59
1:F:106:TYR:CE1	1:F:166:ARG:NH1	2.71	0.58
1:C:160:MET:HG3	3:H:10:EDO:H21	1.85	0.58
1:D:66:ASP:OD1	1:D:68:THR:HG23	2.04	0.57
1:E:74:THR:HG22	1:E:77:GLU:H	1.69	0.57
1:C:166:ARG:NH2	2:G:4:COA:CEP	2.66	0.56
1:F:67:ILE:HG23	1:F:70:PHE:CD2	2.40	0.56
3:H:2:EDO:H11	5:K:722:HOH:O	2.06	0.55
3:H:3:EDO:H21	5:K:760:HOH:O	2.07	0.54
1:C:182:VAL:HG11	1:C:190:GLU:HG3	1.90	0.54
1:C:123:ALA:O	1:C:181:VAL:HA	2.08	0.54
1:E:182:VAL:HG11	1:E:190:GLU:HG3	1.91	0.53
1:B:150[B]:ARG:NH1	5:K:334:HOH:O	2.16	0.53
3:H:10:EDO:C2	5:K:749:HOH:O	2.58	0.52
1:E:10:LYS:HG3	1:E:49:ILE:HD11	1.91	0.52
1:D:129:LEU:N	1:D:129:LEU:HD12	2.25	0.51
1:A:28:LEU:HD23	1:A:32:LEU:HD22	1.92	0.51
1:B:22:PRO:HD2	5:K:696:HOH:O	2.10	0.51
3:H:2:EDO:C1	5:K:722:HOH:O	2.58	0.51
1:C:15:PHE:CD2	1:C:43:ALA:HB2	2.46	0.50
1:C:215:ARG:CZ	1:D:222:LEU:HD23	2.42	0.49
1:C:166:ARG:HG2	5:K:175:HOH:O	2.11	0.49
1:E:166:ARG:NH1	2:G:5:COA:H141	2.27	0.49
1:C:108:LEU:HD12	2:G:4:COA:H131	1.95	0.49
1:F:239:GLU:CD	1:F:239:GLU:H	2.15	0.49
1:A:28:LEU:CD2	1:A:32:LEU:HD22	2.43	0.48
1:A:10:LYS:HE2	5:K:703:HOH:O	2.13	0.48
1:E:19:LEU:HD22	1:E:32:LEU:HD21	1.96	0.48
1:C:231:GLY:HA2	1:D:230:VAL:CG1	2.44	0.48
1:C:230:VAL:CG1	1:D:231:GLY:HA2	2.44	0.48
1:D:40:VAL:HG13	1:D:95:LEU:HD11	1.95	0.48
1:A:236:PHE:CD2	1:A:241:LYS:HE3	2.49	0.48
1:B:36:LEU:O	1:B:40:VAL:HG12	2.14	0.47
1:C:21:ARG:H	1:C:26:ASN:HD22	1.63	0.47

Continued on next page...

PRELIMINARY VALIDATION REPORT

Continued from previous page...

Atom-1	Atom-2	Interatomic distance (Å)	Clash overlap (Å)
1:E:10:LYS:HG3	1:E:49:ILE:CD1	2.45	0.47
1:C:166:ARG:NH2	2:G:4:COA:H141	2.30	0.47
1:D:37:ASP:OD1	1:D:91:LYS:CE	2.63	0.47
1:D:123:ALA:O	1:D:181:VAL:HA	2.15	0.47
1:D:159:GLU:OE2	1:F:180:ARG:NH2	2.48	0.47
1:E:166:ARG:NH1	2:G:5:COA:CEP	2.79	0.46
1:E:201:LYS:NZ	1:F:135:ASN:OD1	2.49	0.46
1:E:5:THR:OG1	1:E:32:LEU:HA	2.16	0.46
1:A:5:THR:OG1	1:A:32:LEU:HA	2.15	0.45
1:F:251:LYS:O	1:F:251:LYS:HG2	2.15	0.45
1:A:123:ALA:O	1:A:181:VAL:HA	2.16	0.45
1:B:175:TYR:HH	4:I:1:GOL:HO1	1.63	0.45
1:D:13:ASN:HB2	1:D:50:ARG:HG3	1.99	0.45
1:F:180:ARG:NH1	1:F:190:GLU:OE1	2.44	0.45
1:D:13:ASN:OD1	3:H:11:EDO:C1	2.64	0.45
1:B:197:LYS:HD2	1:C:163:THR:HB	1.98	0.45
1:A:72:GLN:HG3	5:K:303:HOH:O	2.16	0.45
1:F:106:TYR:HE1	1:F:166:ARG:NH1	2.13	0.44
1:B:40:VAL:HG23	1:B:95:LEU:HD21	1.99	0.44
1:C:230:VAL:HG12	1:D:230:VAL:HG12	1.99	0.44
1:D:50:ARG:HD2	1:D:199:ALA:O	2.18	0.44
1:E:236:PHE:HZ	1:F:70:PHE:HA	1.83	0.44
4:I:5:GOL:H31	5:K:351:HOH:O	2.17	0.44
1:A:230:VAL:CG1	1:F:231:GLY:HA2	2.48	0.44
1:F:123:ALA:O	1:F:181:VAL:HA	2.17	0.43
1:A:214:ASN:HD21	1:B:154:LYS:HZ3	1.66	0.43
1:B:123:ALA:O	1:B:181:VAL:HA	2.18	0.43
1:E:123:ALA:O	1:E:181:VAL:HA	2.19	0.43
1:A:149:THR:HG23	1:A:154:LYS:HA	1.99	0.43
1:A:210:LYS:HD2	1:B:162:MET:CE	2.48	0.43
1:C:149:THR:HG23	1:C:154:LYS:HA	2.01	0.43
1:E:170:LYS:HG3	5:K:543:HOH:O	2.17	0.43
1:C:60:ALA:HB1	1:C:106:TYR:HB2	1.99	0.43
1:E:219:SER:HB3	1:E:220:PRO:HD2	2.01	0.43
1:C:13[B]:ASN:OD1	1:C:14:LEU:HD12	2.19	0.42
1:A:50:ARG:HD3	5:K:828:HOH:O	2.19	0.42
1:B:139:TYR:HB2	1:B:140:PRO:CD	2.49	0.42
1:E:71:ASN:ND2	1:E:72:GLN:HG2	2.34	0.42
1:E:128:GLN:HB3	1:E:166:ARG:HD2	2.01	0.42
1:B:104:ASN:H	1:B:104:ASN:ND2	2.18	0.42
1:C:13[B]:ASN:OD1	1:C:14:LEU:CD1	2.68	0.42

Continued on next page...

PRELIMINARY VALIDATION REPORT

Continued from previous page...

Atom-1	Atom-2	Interatomic distance (Å)	Clash overlap (Å)
1:C:215:ARG:HD3	1:C:215:ARG:HA	1.90	0.41
1:B:241:LYS:O	1:B:245:VAL:HG23	2.21	0.41
1:D:13:ASN:OD1	3:H:11:EDO:H12	2.20	0.41
1:C:11:GLU:OE1	1:C:192:ARG:NH1	2.40	0.41
1:E:34:GLU:O	1:E:38:ARG:HG3	2.21	0.41
1:C:123:ALA:HB3	1:C:181:VAL:HG12	2.02	0.41
1:C:60:ALA:CB	1:C:106:TYR:HB2	2.50	0.41
1:E:158:LEU:HA	1:E:158:LEU:HD23	1.92	0.41
1:A:98:PRO:HA	1:A:119:ASP:OD2	2.21	0.41
1:C:14:LEU:HD12	1:C:14:LEU:N	2.36	0.41
1:A:129:LEU:N	1:A:129:LEU:HD12	2.36	0.41
1:A:150[A]:ARG:HD3	5:K:375:HOH:O	2.20	0.40
1:B:156[A]:ARG:NH1	5:K:306:HOH:O	2.54	0.40
1:C:5:THR:O	1:C:20:ASN:N	2.43	0.40
1:B:215:ARG:HD3	1:B:215:ARG:HA	1.82	0.40
1:B:222:LEU:HA	1:B:222:LEU:HD23	1.94	0.40
1:A:34:GLU:OE1	1:A:38:ARG:NH2	2.55	0.40
1:B:5:THR:OG1	1:B:32:LEU:HA	2.22	0.40
1:D:156:ARG:NH1	1:F:179:ASN:O	2.41	0.40

There are no symmetry-related clashes.

5.3 Torsion angles ⓘ

5.3.1 Protein backbone ⓘ

In the following table, the Percentiles column shows the percent Ramachandran outliers of the chain as a percentile score with respect to all X-ray entries followed by that with respect to entries of similar resolution.

The Analysed column shows the number of residues for which the backbone conformation was analysed, and the total number of residues.

Mol	Chain	Analysed	Favoured	Allowed	Outliers	Percentiles	
1	A	256/259 (99%)	251 (98%)	5 (2%)	0	100	100
1	B	258/259 (100%)	253 (98%)	5 (2%)	0	100	100
1	C	258/259 (100%)	253 (98%)	5 (2%)	0	100	100
1	D	257/259 (99%)	254 (99%)	3 (1%)	0	100	100
1	E	256/259 (99%)	253 (99%)	3 (1%)	0	100	100

Continued on next page...

PRELIMINARY VALIDATION REPORT

Continued from previous page...

Mol	Chain	Analysed	Favoured	Allowed	Outliers	Percentiles	
1	F	255/259 (98%)	252 (99%)	3 (1%)	0	100	100
All	All	1540/1554 (99%)	1516 (98%)	24 (2%)	0	100	100

There are no Ramachandran outliers to report.

5.3.2 Protein sidechains (i)

In the following table, the Percentiles column shows the percent sidechain outliers of the chain as a percentile score with respect to all X-ray entries followed by that with respect to entries of similar resolution.

The Analysed column shows the number of residues for which the sidechain conformation was analysed, and the total number of residues.

Mol	Chain	Analysed	Rotameric	Outliers	Percentiles	
1	A	209/209 (100%)	206 (99%)	3 (1%)	69	62
1	B	211/209 (101%)	207 (98%)	4 (2%)	60	49
1	C	210/209 (100%)	207 (99%)	3 (1%)	69	62
1	D	209/209 (100%)	204 (98%)	5 (2%)	52	38
1	E	209/209 (100%)	206 (99%)	3 (1%)	69	62
1	F	208/209 (100%)	203 (98%)	5 (2%)	52	38
All	All	1256/1254 (100%)	1233 (98%)	23 (2%)	62	51

All (23) residues with a non-rotameric sidechain are listed below:

Mol	Chain	Res	Type
1	A	179	ASN
1	A	222	LEU
1	A	232	TRP
1	B	59	LYS
1	B	232	TRP
1	B	241	LYS
1	B	249	LEU
1	C	218	ASP
1	C	232	TRP
1	C	241	LYS
1	D	166	ARG
1	D	179	ASN
1	D	218	ASP

Continued on next page...

PRELIMINARY VALIDATION REPORT

Continued from previous page...

Mol	Chain	Res	Type
1	D	232	TRP
1	D	246	SER
1	E	57	LYS
1	E	74	THR
1	E	218	ASP
1	F	66	ASP
1	F	218	ASP
1	F	222	LEU
1	F	236	PHE
1	F	253	GLU

Some sidechains can be flipped to improve hydrogen bonding and reduce clashes. All (17) such sidechains are listed below:

Mol	Chain	Res	Type
1	A	72	GLN
1	A	179	ASN
1	A	214	ASN
1	B	71	ASN
1	B	104	ASN
1	C	26	ASN
1	C	186	ASN
1	D	42	GLN
1	D	179	ASN
1	D	189	GLN
1	E	13	ASN
1	E	26	ASN
1	E	71	ASN
1	E	72	GLN
1	E	128	GLN
1	E	179	ASN
1	F	179	ASN

5.3.3 RNA (i)

There are no RNA molecules in this entry.

5.4 Non-standard residues in protein, DNA, RNA chains (i)

There are no non-standard protein/DNA/RNA residues in this entry.

PRELIMINARY VALIDATION REPORT

5.5 Carbohydrates (i)

There are no carbohydrates in this entry.

5.6 Ligand geometry (i)

25 ligands are modelled in this entry.

In the following table, the Counts columns list the number of bonds (or angles) for which Mogul statistics could be retrieved, the number of bonds (or angles) that are observed in the model and the number of bonds (or angles) that are defined in the Chemical Component Dictionary. The Link column lists molecule types, if any, to which the group is linked. The Z score for a bond length (or angle) is the number of standard deviations the observed value is removed from the expected value. A bond length (or angle) with $|Z| > 2$ is considered an outlier worth inspection. RMSZ is the root-mean-square of all Z scores of the bond lengths (or angles).

Mol	Type	Chain	Res	Link	Bond lengths			Bond angles		
					Counts	RMSZ	$\#\|Z\| > 2$	Counts	RMSZ	$\#\|Z\| > 2$
2	COA	G	2	-	41,50,50	1.06	3 (7%)	51,75,75	1.66	8 (15%)
2	COA	G	3	-	41,50,50	1.04	3 (7%)	51,75,75	3.03	9 (17%)
2	COA	G	4	-	41,50,50	0.95	2 (4%)	51,75,75	1.62	7 (13%)
2	COA	G	5	-	41,50,50	0.91	2 (4%)	51,75,75	1.70	7 (13%)
3	EDO	H	10	-	3,3,3	0.42	0	2,2,2	0.42	0
3	EDO	H	11	-	3,3,3	0.40	0	2,2,2	0.30	0
3	EDO	H	12	-	3,3,3	0.50	0	2,2,2	0.53	0
3	EDO	H	13	-	3,3,3	0.42	0	2,2,2	0.50	0
3	EDO	H	2	-	3,3,3	0.72	0	2,2,2	0.52	0
3	EDO	H	3	-	3,3,3	0.85	0	2,2,2	0.65	0
3	EDO	H	4	-	3,3,3	0.38	0	2,2,2	0.31	0
3	EDO	H	5	-	3,3,3	0.62	0	2,2,2	0.42	0
3	EDO	H	7	-	3,3,3	0.70	0	2,2,2	0.15	0
3	EDO	H	8	-	3,3,3	0.38	0	2,2,2	0.26	0
3	EDO	H	9	-	3,3,3	0.54	0	2,2,2	0.11	0
4	GOL	I	1	-	5,5,5	0.54	0	5,5,5	0.94	0
4	GOL	I	10	-	5,5,5	0.48	0	5,5,5	0.81	0
4	GOL	I	11	-	5,5,5	0.24	0	5,5,5	0.27	0
4	GOL	I	2	-	5,5,5	0.55	0	5,5,5	0.45	0
4	GOL	I	3	-	5,5,5	0.55	0	5,5,5	0.66	0
4	GOL	I	4	-	5,5,5	0.60	0	5,5,5	0.47	0
4	GOL	I	5	-	5,5,5	0.63	0	5,5,5	1.37	1 (20%)
4	GOL	I	6	-	5,5,5	0.46	0	5,5,5	0.48	0
4	GOL	I	7	-	5,5,5	0.41	0	5,5,5	0.63	0
4	GOL	I	9	-	5,5,5	0.33	0	5,5,5	0.61	0

PRELIMINARY VALIDATION REPORT

In the following table, the Chirals column lists the number of chiral outliers, the number of chiral centers analysed, the number of these observed in the model and the number defined in the Chemical Component Dictionary. Similar counts are reported in the Torsion and Rings columns. '-' means no outliers of that kind were identified.

Mol	Type	Chain	Res	Link	Chirals	Torsions	Rings
2	COA	G	2	-	-	0/44/64/64	0/3/3/3
2	COA	G	3	-	-	0/44/64/64	0/3/3/3
2	COA	G	4	-	-	0/44/64/64	0/3/3/3
2	COA	G	5	-	-	0/44/64/64	0/3/3/3
3	EDO	H	10	-	-	0/1/1/1	0/0/0/0
3	EDO	H	11	-	-	0/1/1/1	0/0/0/0
3	EDO	H	12	-	-	0/1/1/1	0/0/0/0
3	EDO	H	13	-	-	0/1/1/1	0/0/0/0
3	EDO	H	2	-	-	0/1/1/1	0/0/0/0
3	EDO	H	3	-	-	0/1/1/1	0/0/0/0
3	EDO	H	4	-	-	0/1/1/1	0/0/0/0
3	EDO	H	5	-	-	0/1/1/1	0/0/0/0
3	EDO	H	7	-	-	0/1/1/1	0/0/0/0
3	EDO	H	8	-	-	0/1/1/1	0/0/0/0
3	EDO	H	9	-	-	0/1/1/1	0/0/0/0
4	GOL	I	1	-	-	0/4/4/4	0/0/0/0
4	GOL	I	10	-	-	0/4/4/4	0/0/0/0
4	GOL	I	11	-	-	0/4/4/4	0/0/0/0
4	GOL	I	2	-	-	0/4/4/4	0/0/0/0
4	GOL	I	3	-	-	0/4/4/4	0/0/0/0
4	GOL	I	4	-	-	0/4/4/4	0/0/0/0
4	GOL	I	5	-	-	0/4/4/4	0/0/0/0
4	GOL	I	6	-	-	0/4/4/4	0/0/0/0
4	GOL	I	7	-	-	0/4/4/4	0/0/0/0
4	GOL	I	9	-	-	0/4/4/4	0/0/0/0

All (10) bond length outliers are listed below:

Mol	Chain	Res	Type	Atoms	Z	Observed(Å)	Ideal(Å)
2	G	4	COA	C2A-N3A	2.03	1.35	1.32
2	G	3	COA	O4B-C1B	2.12	1.44	1.41
2	G	3	COA	C2A-N3A	2.13	1.35	1.32
2	G	2	COA	C2A-N1A	2.17	1.38	1.33
2	G	5	COA	O4B-C1B	2.31	1.44	1.41
2	G	5	COA	C5A-C4A	3.17	1.47	1.40
2	G	3	COA	C5A-C4A	3.24	1.47	1.40
2	G	2	COA	C5A-C4A	3.26	1.47	1.40
2	G	4	COA	C5A-C4A	3.28	1.47	1.40
2	G	2	COA	P3B-O3B	3.32	1.65	1.59

PRELIMINARY VALIDATION REPORT

All (32) bond angle outliers are listed below:

Mol	Chain	Res	Type	Atoms	Z	Observed(^{o})	Ideal(^{o})
2	G	3	COA	CEP-CBP-CAP	-11.72	88.49	108.82
2	G	3	COA	CDP-CBP-CAP	-9.77	91.89	108.82
2	G	5	COA	N3A-C2A-N1A	-7.61	122.35	128.86
2	G	2	COA	N3A-C2A-N1A	-7.49	122.45	128.86
2	G	3	COA	N3A-C2A-N1A	-7.40	122.53	128.86
2	G	4	COA	N3A-C2A-N1A	-6.69	123.14	128.86
2	G	5	COA	C7P-N8P-C9P	-3.66	115.87	122.59
2	G	4	COA	C4A-C5A-N7A	-3.40	106.13	109.41
2	G	2	COA	P2A-O3A-P1A	-3.28	121.59	132.63
2	G	3	COA	P2A-O3A-P1A	-3.09	122.23	132.63
2	G	5	COA	C6P-C7P-N8P	-2.80	106.17	111.85
2	G	5	COA	P2A-O3A-P1A	-2.66	123.69	132.63
2	G	2	COA	O3B-P3B-O7A	-2.52	99.65	109.39
4	I	5	GOL	C3-C2-C1	-2.52	101.93	111.63
2	G	2	COA	C1B-N9A-C4A	-2.37	122.54	126.64
2	G	3	COA	C4A-C5A-N7A	-2.37	107.12	109.41
2	G	4	COA	O5P-C5P-C6P	-2.30	117.73	122.00
2	G	4	COA	P2A-O3A-P1A	-2.15	125.39	132.63
2	G	5	COA	O5P-C5P-C6P	-2.03	118.23	122.00
2	G	4	COA	C6P-C7P-N8P	2.03	115.98	111.85
2	G	5	COA	N6A-C6A-N1A	2.07	122.87	118.57
2	G	2	COA	C2A-N1A-C6A	2.25	122.58	118.75
2	G	2	COA	O2B-C2B-C3B	2.33	117.77	111.17
2	G	5	COA	C2A-N1A-C6A	2.92	123.71	118.75
2	G	2	COA	O5A-P2A-O4A	3.05	127.64	112.14
2	G	4	COA	C3P-N4P-C5P	3.09	128.71	122.85
2	G	3	COA	O6A-CCP-CBP	3.16	115.62	110.55
2	G	2	COA	O9A-P3B-O8A	3.17	120.12	107.59
2	G	4	COA	C6P-C5P-N4P	3.26	122.11	116.46
2	G	3	COA	CDP-CBP-CCP	5.52	117.23	108.23
2	G	3	COA	CEP-CBP-CCP	5.72	117.56	108.23
2	G	3	COA	CEP-CBP-CDP	7.56	124.94	109.16

There are no chirality outliers.

There are no torsion outliers.

There are no ring outliers.

8 monomers are involved in 16 short contacts:

Mol	Chain	Res	Type	Clashes	Symm-Clashes
2	G	4	COA	5	0

Continued on next page...

PRELIMINARY VALIDATION REPORT

Continued from previous page...

Mol	Chain	Res	Type	Clashes	Symm-Clashes
2	G	5	COA	2	0
3	H	10	EDO	2	0
3	H	11	EDO	2	0
3	H	2	EDO	2	0
3	H	3	EDO	1	0
4	I	1	GOL	1	0
4	I	5	GOL	1	0

5.7 Other polymers (i)

There are no such residues in this entry.

5.8 Polymer linkage issues (i)

There are no chain breaks in this entry.

PRELIMINARY VALIDATION REPORT

6 Fit of model and data (i)

6.1 Protein, DNA and RNA chains (i)

In the following table, the column labelled '#RSRZ> 2' contains the number (and percentage) of RSRZ outliers, followed by percent RSRZ outliers for the chain as percentile scores relative to all X-ray entries and entries of similar resolution. The OWAB column contains the minimum, median, 95th percentile and maximum values of the occupancy-weighted average B-factor per residue. The column labelled 'Q< 0.9' lists the number of (and percentage) of residues with an average occupancy less than 0.9.

Mol	Chain	Analysed	<RSRZ>	#RSRZ>2			OWAB(Å^2)	Q<0.9
1	A	257/259 (99%)	-0.16	6 (2%)	60	56	11, 18, 46, 75	0
1	B	257/259 (99%)	-0.00	9 (3%)	44	38	13, 23, 52, 76	0
1	C	259/259 (100%)	0.06	7 (2%)	54	49	10, 25, 53, 82	0
1	D	258/259 (99%)	-0.16	9 (3%)	44	38	11, 19, 49, 72	0
1	E	257/259 (99%)	0.06	6 (2%)	60	56	12, 27, 49, 83	0
1	F	257/259 (99%)	0.09	16 (6%)	20	16	11, 26, 59, 77	0
All	All	1545/1554 (99%)	-0.02	53 (3%)	45	40	10, 23, 52, 83	0

All (53) RSRZ outliers are listed below:

Mol	Chain	Res	Type	RSRZ
1	E	1	MET	9.0
1	C	1	MET	7.0
1	A	1	MET	5.6
1	F	1	MET	5.5
1	D	1	MET	5.1
1	F	71	ASN	4.9
1	B	257	LYS	4.8
1	F	72	GLN	4.7
1	B	249	LEU	4.7
1	C	259	LYS	4.5
1	C	72	GLN	4.5
1	D	71	ASN	4.3
1	B	1	MET	4.2
1	A	253	GLU	3.9
1	E	72	GLN	3.7
1	F	249	LEU	3.7
1	B	251	LYS	3.6
1	F	251	LYS	3.5
1	F	68	THR	3.5

Continued on next page...

PRELIMINARY VALIDATION REPORT

Continued from previous page...

Mol	Chain	Res	Type	RSRZ
1	B	72	GLN	3.4
1	F	255	THR	3.4
1	F	23	ASP	3.4
1	D	2	GLU	3.3
1	C	71	ASN	3.2
1	D	257	LYS	3.1
1	C	23	ASP	3.0
1	B	253	GLU	3.0
1	D	258	GLY	3.0
1	F	257	LYS	2.9
1	A	252	ARG	2.8
1	A	251	LYS	2.8
1	F	253	GLU	2.8
1	A	257	LYS	2.7
1	C	2	GLU	2.7
1	E	23	ASP	2.7
1	B	2	GLU	2.7
1	E	251	LYS	2.7
1	B	250	GLU	2.5
1	F	252	ARG	2.5
1	F	69	GLN	2.4
1	D	23	ASP	2.4
1	D	72	GLN	2.4
1	D	253	GLU	2.3
1	D	69	GLN	2.3
1	F	248	PHE	2.2
1	B	248	PHE	2.2
1	C	38	ARG	2.2
1	F	247	ALA	2.2
1	A	242	LYS	2.2
1	F	246	SER	2.1
1	F	59	LYS	2.1
1	E	250	GLU	2.1
1	E	257	LYS	2.1

6.2 Non-standard residues in protein, DNA, RNA chains (i)

There are no non-standard protein/DNA/RNA residues in this entry.

PRELIMINARY VALIDATION REPORT

6.3 Carbohydrates (i)

There are no carbohydrates in this entry.

6.4 Ligands (i)

In the following table, the Atoms column lists the number of modelled atoms in the group and the number defined in the chemical component dictionary. The B-factors column lists the minimum, median, 95th percentile and maximum values of B factors of atoms in the group. The column labelled 'Q< 0.9' lists the number of atoms with occupancy less than 0.9.

Mol	Type	Chain	Res	Atoms	RSCC	RSR	B-factors(Å^2)	Q<0.9
2	COA	G	3	48/?	0.75	0.23	57,68,80,84	0
2	COA	G	5	48/?	0.75	0.24	67,80,88,96	0
3	EDO	H	7	4/?	0.76	0.14	47,50,50,50	0
4	GOL	I	11	6/?	0.77	0.17	53,55,56,57	0
2	COA	G	4	48/?	0.77	0.26	60,74,83,85	0
4	GOL	I	6	6/?	0.80	0.14	35,40,41,43	0
4	GOL	I	5	6/?	0.83	0.14	41,42,44,45	0
4	GOL	I	3	6/?	0.85	0.13	38,43,45,46	0
3	EDO	H	4	4/?	0.86	0.15	47,47,50,51	0
3	EDO	H	3	4/?	0.87	0.10	33,33,36,37	0
4	GOL	I	4	6/?	0.87	0.13	26,30,34,40	0
4	GOL	I	10	6/?	0.87	0.12	42,45,50,51	0
4	GOL	I	1	6/?	0.88	0.13	41,47,47,48	0
4	GOL	I	2	6/?	0.89	0.12	40,43,44,46	0
4	GOL	I	7	6/?	0.90	0.10	42,44,45,46	0
3	EDO	H	5	4/?	0.90	0.15	39,39,41,41	0
3	EDO	H	9	4/?	0.91	0.14	30,33,37,42	0
4	GOL	I	9	6/?	0.92	0.13	38,45,46,47	0
3	EDO	H	13	4/?	0.92	0.21	41,43,45,47	0
3	EDO	H	8	4/?	0.92	0.09	29,33,35,38	0
3	EDO	H	12	4/?	0.93	0.10	31,33,37,41	0
3	EDO	H	2	4/?	0.94	0.10	24,27,28,30	0
3	EDO	H	11	4/?	0.95	0.20	40,43,44,46	0
3	EDO	H	10	4/?	0.96	0.12	36,37,37,40	0
2	COA	G	2	48/?	0.97	0.07	16,23,27,37	0

6.5 Other polymers (i)

There are no such residues in this entry.

PRELIMINARY VALIDATION REPORT
